# Multifunctional role of dietary copper to regulate stress-responsive gene for mitigation of multiple stresses in *Pangasianodon hypophthalmus*

**DOI:** 10.1038/s41598-024-51170-z

**Published:** 2024-01-26

**Authors:** Neeraj Kumar, Supriya Tukaram Thorat, Samiksha R. Chavhan

**Affiliations:** ICAR-National Institute of Abiotic Stress Management, Baramati, Pune, Maharashtra 413115 India

**Keywords:** Animal physiology, Ichthyology

## Abstract

It is an urgent needs to address climate change and pollution in aquatic systems using suitable mitigation measures to avoid the aquatic animals' extinction. The vulnerability and extinction of the aquatic animals in the current scenario must be addressed to enhance safe fish food production. Taking into consideration of such issues in fisheries and aquaculture, an experiment was designed to mitigate high temperature (T) and low pH stress, as well as arsenic (As) pollution in fish using copper (Cu) containing diets. In the present investigation, the Cu-containing diets graded with 0, 4, 8, and 12 mg kg^-1^ were prepared and fed to *Pangasianodon hypophthalmus* reared under As, low pH, and high-temperature stress. The gene expression was highly affected in terms of the primary, secondary, and tertiary stress response, whereas supplementation of Cu-containing diet mitigates the stress response. Oxidative stress genes such as catalase (*CAT*), superoxide dismutase (*SOD*), and glutathione peroxidase (*GPx*) were significantly upregulated by stressors (As, As + T, and As + pH + T). Whereas, heat shock protein (*HSP 70*), inducible nitric oxide synthase (*iNOS*), metallothionine (*MT*), caspase 3a (*Cas 3a*), and cytochrome P450 (*CYP 450*) were highly upregulated by stressors, while dietary Cu at 8 mg kg^-1^ diet significantly downregulated these gene expressions. Indeed, the immunity-related genes viz. *TNFα, Ig, TLR*, and immune-related attributes viz. albumin, globulin, total protein, A:G ratio, blood glucose, NBT, and myeloperoxidase (MPO) were also improved with Cu-containing diets. Cu containing diets substantially improved neurotransmitter enzyme (AChE) and vitamin C (Vit C). DNA damage was also reduced with supplementation of Cu at 8 mg kg^-1^ diet. The growth index viz. final body weight gain (%), specific growth rate, protein efficiency ratio, food conversion ratio, relative feed intake, and daily growth index were noticeably enhanced by Cu diets (4 and 8 mg kg^-1^ diet). The growth-related genes expressions viz. growth hormone (*GH*)*,* growth hormone regulator 1 (*Ghr1),* growth hormone regulator β (*Ghrβ,*) *myostatin* (*MYST*), and somatostatin (*SMT*) supported the growth enhancement with Cu at 8 mg kg^-1^ diet. The bioaccumulation of As was reduced with Cu-containing diets. The fish were infected with *Aeromonas hydrophila* at the end of the 105 days experimental trial. Cu at 8 mg kg^-1^ diet improved immunity, reduced the cumulative mortality, and enhanced the relative percentage survival of the fish. The results revealed that the innovative Cu diets could reduce the extinction of the fish against climate change and pollution era and produce the safest production that is safe to humans for consumption.

## Introduction

The aquatic ecosystem is threatened daily by climate change, and pollution all over the globe is unequivocal^1^. Temperature fluctuations from higher to lower and vice versa are depicted in the aquatic system and affect aquatic organisms, including fish^2^. The pollution and temperature fluctuation affect aquatic systems, degrade biodiversity, cause the extinction of fish species, change feeding habits, bioaccumulation of contaminants, and several other deformities occur in the system. The water quality of aquatic systems also varies due to pollution and temperature. The pH is the most limiting factor in aquaculture and fisheries. It is also affected by temperature change, dissolved oxygen, organic decomposition, respiration by aquatic animals, and photosynthesis in aquatic systems^3^. The lower pH enhances toxic metal release in aquaculture and fisheries^4,5^. The pH is a very sensitive water quality parameter, as a small change in pH results in mass mortality of aquatic animals^6^. The toxicity of arsenic is enhanced with low pH and high temperature and induces stress in fish. Generally, many stressors, like abiotic and biotic factors, are present in the aquatic system, enhancing the stress response in aquatic animals. Indeed, this study addresses the effect of multiple abiotic and biotic factors such as high temperature (34 °C), low pH (6.5), arsenic toxicity and pathogenic infection in fish. Arsenic is widely used for agriculture, veterinary drugs, medicines, metal alloy manufacturing, microelectronics, glassware, and wood preservatives^7,8^. As per the International Agency for Research on Cancer (IARC), arsenic is considered Class I carcinogenic^9^. It is a very dangerous metalloid affecting 300 million people worldwide, including Asian countries^10^. Acute and chronic exposure of arsenic is lethal to all living organisms, including humans, animals, and fish^2,11^. The intake of arsenic causes keratosis, melanosis, hyperpigmentation, cancer, and other serious diseases in humans, animals and fish^2,12–15^.

Copper is a crucial and essential element for humans, animals, and fish^16^. It is also important for several biological processes viz. formation of bone, synthesis of hemoglobin, nervous system to maintain the myelin sheet, and co-factor for several enzymes such as dopamine hydroxylase, superoxide dismutase, cytochrome oxidase, tyrosinase and ferroxidase^17–19^. Cu acts as growth promoting agent, metabolism, immunomodulator, and anti-oxidant in fish^20–24^. It also plays an essential role in iron metabolism as a component of ceruloplasmin, circulating in the blood plasma and bound to Cu. It showed the ferroxidase activity necessary for iron circulation^25,26^. Taking into consideration, it should be used in optimum quantity; excess use of Cu may damage the tissues and organs as it forms a complex and initiates the process for reactive oxygen species (ROS) and damage the DNA, lipid, and protein^27,28^. It also protects the cells against oxidative damage, ceruloplasmin, and metallothioneins^29^.

The gene expressions of superoxide dismutase (*SOD*), catalase (*CAT*), glutathione peroxidase (*GPx*), heat shock protein (*HSP*), inducible nitric oxide synthase (*iNOS*), metallothionine (*MT*), , tumour necrosis factor (*TNFα*), toll like receptor (*TLR*), total immunoglobulin (*Ig*), growth hormone (*GH*), growth hormone regulator 1 (*GHR1*), growth hormone regulator β (*GHRβ*), myostatin (*MYST*), and somatostatin (*SMT*) were highly affected by arsenic pollution, low pH and high temperature. These gene expressions are stress-responsive in *P. hypophthalmus*. Similarly, Cu has an essential role in immunomodulation in fish as it enhances immunity against stress and pathogenic bacterial infection in fish^30,31^. In the present investigation, nuclear factor-kappa B (NF-κB) has been addressed to understand the mechanism of Cu protection against multiple stresses.

*P. hypophthalmus* is a potential candidate species for culturing in the abiotic and biotically stressed zone^32–34^. It has a trait of diversification, high growth rate, sturdy, high demand, medicinal value, and is suited for intensive culture^2,12,13^. Although, this species has yet to receive much research attention worldwide to multiple stresses. The present study deals with mitigating arsenic pollution, low pH, and high-temperature stress using different graded levels of dietary copper. This investigation also deals with identifying stress-responsive genes and their regulation during exposure to multiple stress (As + pH + T) and the role of copper in regulating the gene expressions for mitigating abiotic and biotic stress in *P. hypophthalmus.*

## Material and methods

### Ethics statement

The National Institute of Abiotic Stress Management, Research Advisory Committee (RAC) and Director of the Institute has approved the experimental procedures. The present study also compliance with Animal Research: Reporting of In Vivo Experiments (ARRIVE) guidelines. The approval was obtained from the institute PME as 7-1(PME) 2012-342.

### Experimental design and diet preparation

*P. hypophthalmus* was obtained from the fish farmer at Sangali, Maharashtra, India. The fish were stocked in the NIASM farm pond for acclimatization for 2 months and fed with 30% proteaceous diet. The rectangular plastic tank was used for an experiment with a capacity of 150 L. The tanks were cleaned and disinfected with KMnO_4_ (2 ppm) and salt (1%). Before the commencement of the experiment, the *P. hypophthalmus* (weight: 5.78 ± 0.28 g and length: 5.34 cm) was again acclimatized in the plastic rectangular tank for 7 days. The experiment was designed with 10 treatments in three replicates, and eighteen fish (18) were used in each replicate, so a total of five hundred forty fish were used for this experiment. The fish were treated under different abiotic stress conditions such as exposure to arsenic, low pH (6.5), and high temperature (34 °C) with different combinations. The Cu-containing diets (0, 4, 8, 12 mg kg^-1^ diet) were formulated and used to mitigate multiple stressors (As + pH + T). The details of the experimental design and treatments are shown in Table 1. Water quality was periodically recorded till the end of the 105 days experiment (American Public Health Association, APHA)^35^ (Supp Table 1). The 2/3rd of the water was manually replaced every second day, and by adding, the concentration of arsenic was maintained in experimental water. The stressors groups (As, As + pH and As + pH + T) was maintained via*.* As (1/10th of LC_50_ 2.68 mg L^-1^ of arsenic)^2^, pH (6.5) and high temperature (34 °C) were maintained with a thermostatic heater. The pH of the experimental water was maintained 6.5 using 0.1 N HCl or 0.1 N NaOH and phosphate buffer (0.1 M for pH 6.5) to maintain constant pH during 105 days experiment^36–38^. Moreover, the pH was monitored thrice every day by a digital pH meter. Experimental diets were provided to fish, and uneaten diet and faecal matter were removed by siphoning in each tank daily. A compressed air pump was provided for continuous aeration. The four experimental diets of iso-nitrogenous (35% crude protein) and iso-caloric (393 kcal/100 g) were prepared. The different feed ingredients were used as fish meal, groundnut meal, soybean meal, wheat flour, carboxymethyl cellulose (CMC), cod liver oil, lecithin, and vitamin C. The copper-free vitamin-mineral mixture was prepared manually for inclusion in the diet. The heat-labile ingredients were mixed after heating the feed ingredient. Proximate analysis of the diets was also analyzed using AOAC method^39^. Crude protein was analyzed using nitrogen content, ether extract (EE) using solvent extraction, and Ash estimation using a muffle furnace (550 °C) (Table 2). Total carbohydrate% was calculated using the following equation:

$${\text{Total}}\;{\text{ carbohydrate}}\% = {1}00 - \left( {{\text{Moisture}} + {\text{CP}}\% + {\text{ EE}}\% \, + {\text{Ash}}\% } \right).$$

**Table 1:** Details of experimental design.

Treatment number (s)	Treatments	Treatments details	Symbols
1	Control group	No exposure to stressors (arsenic + low pH + high temperature (34 °C, and no supplementation of Cu) and fed with control diet	Control
2	Arsenic (2.68 mg L^-1^)	Exposure to arsenic and fed with control diet	As
3	Arsenic (2.68 mg L^-1^) and high temperature (34 °C) exposure	Concurrent exposure to arsenic (2.68 mg L^-1^) and high temperature (34 °C) and fed with control diet	As + T
4	Arsenic (2.68 mg L^-1^), low pH (6.5) and high temperature (34 °C) exposure	Concurrent exposure to arsenic (2.68 mg L^-1^), low pH (6.5) and high temperature (34 °C) and fed with control diet	As + T + pH
5	Cu at 4 mg kg^-1^ diet	Fed with Cu at 4.0 mg kg^-1^	Cu-4.0 mg kg^-1^
6	Cu at 8 mg kg^-1^ diet	Fed with Cu at 8.0 mg kg^-1^	Cu-8.0 mg kg^-1^
7	Cu at 12 mg kg^-1^ diet	Fed with Cu at 12.0 mg kg^-1^	Cu-12.0 mg kg^-1^
8	Cu at 4 mg kg^-1^ diet and exposure to stressors (As + pH + T)	Fed with Cu at 4.0 mg kg^-1^ and concurrent exposure to arsenic (2.68 mg L^-1^), low pH (6.5) and high temperature (34 °C)	Cu-4.0 mg kg^-1^As + pH + T
9	Cu at 8 mg kg^-1^ diet and exposure to stressors (As + pH + T)	Fed with Cu at 8 mg kg^-1^ diet and treated under concurrent exposure to arsenic (2.68 mg L^-1^), low pH (6.5) and high temperature (34 °C)	Cu-8.0 mg kg^-1^ + As + pH + T
10	Cu at 12 mg kg^-1^ diet and exposure to stressors (As + pH + T)	Fed with Cu at 12.0 mg kg^-1^ diet and treated under concurrent exposure to arsenic (2.68 mg L^-1^), low pH (6.5) and high temperature (34 °C)	Cu-12.0 mg kg^-1^ + As + pH + T

**Table 2:** Ingredient composition and proximate analysis of experimental diets (% dry matter) of copper (Cu), fed to *Pangasianodon hypophthalmus* during the experimental period of 105 days.

Ingredients	Control diet	Copper diets		
		Cu-4.0 mg kg^-1^	Cu-8.0 mg kg^-1^	Cu-12.0 mg kg^-1^
Soybean meal^a^	35.5	35.5	35.5	35.5
Fish meal^a^	20.0	20.0	20.0	20.0
Groundnut meal^a^	10.0	10.0	10.0	10.0
Wheat flour^a^	22.47	22.4696	22.4692	22.4688
Sunflower oil	4.5	4.5	4.5	4.5
Cod liver oil^a^	1.5	1.5	1.5	1.5
CMC^b^	2.0	2.0	2.0	2.0
Vitamin and mineral mix*	2.0	2.0	2.0	2.0
Lecithin	2.0	2.0	2.0	2.0
Vitamin C^d^	0.03	0.03	0.03	0.03
Copper	0.0	0.00040	0.0008	0.0012
Proximate composition of the diets				
CP^1^	35.09 ± 0.11	35.38 ± 0.20	35.34 ± 0.30	35.24 ± 0.03
EE^2^	8.71 ± 0.18	9.05 ± 0.08	8.99 ± 0.06	9.12 ± 0.01
Ash	8.15 ± 0.10	8.34 ± 0.16	8.33 ± 0.12	8.47 ± 0.07
TC^3^	48.05 ± 0.40	47.23 ± 0.24	47.34 ± 0.29	47.17 ± 0.34
OM^4^	91.85 ± 0.09	91.66 ± 0.04	91.67 ± 0.09	91.53 ± 0.09
DM^5^	92.46 ± 0.22	92.16 ± 0.23	92.31 ± 0.19	92.10 ± 0.29
DE^6^	393.53 ± 0.42	393.81 ± 0.36	393.67 ± 0.22	393.48 ± 0.35

^a^Procured from local market, ^b^Himedia Ltd, c*Prepared manually and all components from Himedia Ltd, cSD Fine Chemicals Ltd., India.

*Manual prepared Vitamin mineral mixture; Composition of vitamin mineral mix (quantity/250 g starch powder): vitamin A 55,00,00 IU; vitamin D3 11,00,00 IU; vitamin B1:20 mg; vitamin E 75 mg; vitamin K 1,00 mg; vitamin B12 0.6 mcg; calcium pantothenate 2,50 mg; nicotinamide 1000 mg; pyridoxine: 100 mg; Mn 2700 mg; I 1,00 mg; Fe 750 mg; Co 45 mg; Ca 50 g; P 30 g; Zn 5,00 mg; Se: 2 ppm.

CP^1^- Crude Protein; EE^2^- Ether extract; TC^3^-Total Carbohydrate; OM^4^-Organic Matter, DM^5^-Dry matter, DE^6^- Digestible Energy.

Digestible energy (DE) (Kcal/100 g) = (% CP × 4) + (% EE × 9) + (TC × 4).

Data expressed as mean ± SE, *n* = 3.

The diets' gross energy was calculated using Halver’s method^40^.

### Tissue homogenate preparation and blood collection

During dissection, the gill, kidney, liver, and brain tissues were collected from anesthetized fish (clove oil, 100 µl L^-1^). The chilled sucrose (5% w/v, 0.25 M) and EDTA solution (1 mM) were used for homogenization (Omni Tissue Master Homogenize, Kennesaw, GA). The homogenate samples were centrifuged at 5000 × g at 4 °C for 15 min in a cooling centrifuge (Eppendorf AG, 5430R, Hamburg, Germany), and then samples were stored at − 80 °C until further analysis. The four fish were used for serum (heparin-free syringe), and 3 fish were used for blood with a heparin syringe to avoid blood clotting. Lowry method^41^ was used for the determination of tissue protein.

### RNA isolation and quantification

Total RNA was isolated from the liver tissue of *P. hypophthalmus* using TRIZOL reagent (Catalogue no. 15596018; Invitrogen™, Life Technologies Corporation, Carlsbad, California 92008, USA). Liver tissue of 50 mg homogenized in liquid nitrogen using a mortar pestle and lysed in TRIzol reagent. It was incubated for 5 min after adding chloroform for phase separation. After centrifugation, the aqueous phase containing RNA separated into 1.5 ml tube, and the RNA precipitated using isopropanol. The precipitated RNA was washed with 75% ethanol, and air-dried RNA pellet was dissolved in RNAse free water. The RNA was stored at − 80 °C for further use. RNA integrity was verified by 1.0% agarose gel. It was prepared by melting the required amount of agarose in 1X TAE buffer. The RNA bands were visualized in a gel documentation system (ChemiDocTM MP imaging system, Bio-Rad). The RNA was quantified using a Nano-Drop spectrophotometer (Thermo-scientific)^42^.

### cDNA synthesis and quantitative PCR

Total isolated RNA was used for cDNA synthesis using Revert Aid First strand cDNA synthesis kit (Catalog number, K1622, Thermo Fisher Scientific Baltics UAB, Lithuania, Europe). DNase I was used to remove trace amounts of DNA before cDNA synthesis. The reaction mixture of RNA (100 ng) and oligo dT primers (15 pmol) was placed in 12 µl. The reaction mixture was heated at 65 °C for 5 min in PCR and then chilled on ice. Then 1 µl Ribo Lock RNase Inhibitor (20 U/µL), 1.0 µl of reverse transcriptase enzyme, 5 X reaction buffer (4.0 µl), and 2 µl dNTP Mix (10 mM) were added to the chilled mixture, followed by centrifuge for few second. After that, the mixture was incubated at 60 °C for 42 min and then at 70 °C for 5 min and stored the synthesized cDNA at -20 °C. The synthesized cDNA was confirmed using β-actin PCR. Gene-specific primers were used to perform quantitative PCR (Real-time PCR) using SYBR green PCA master mix (Catalog number A25742, Bio-Rad, UAB, Lithuania, Europe). The samples for quantification containing SYBR Green Master Mix (1X), primer (1 µl) and 1 µl of cDNA and set up for the reaction cycle as Initial denaturation at 95 °C for 10 min, amplification of the cDNA for 39 cycles and then denaturation for 15 s at 95 °C and annealing for 1 min at 60°C^42^. The details of the primers are mentioned in the Table 3. The PCR efficiency and standard curve are presented in Supp Table 2 and Supp Figs. 1, 2, 3. 2-ΔΔCT method was used for calculating relative quantification as per method of Pfaffl^42^.

$$\Delta \Delta {\text{CT}} = \Delta {\text{CT}}\left( {{\text{a}}\;{\text{target}}\;{\text{sample}}} \right) - \Delta {\text{CT}}\left( {{\text{a}}\;{\text{reference}}\;{\text{sample}}} \right) = \left( {{\text{CTD}} - {\text{CTB}}} \right) - \left( {{\text{CTC}} - {\text{CTA}}} \right).$$

whereas,

$$\Delta {\text{CT}} = {\text{CT}}\left( {{\text{a}}\;{\text{ target }}\;{\text{gene}}} \right) - {\text{CT}}\left( {{\text{a}}\;{\text{ reference }}\;{\text{gene}}} \right).$$

the Δ*CT* for the target sample is *CT*_*D*_ − *CT*_*B*_, and the Δ*CT* for the reference sample is *CTC − CTA*.

**Table 3:** Details of primer for relative quantitative real-time PCR.

Gene	Primer sequence (5′ –3′)	Accession number
*SOD*	F-GTCCATCTTACCCGGTGCCC R-CGAGAGAAGACCCGGAACGC	XM_034299545.1
*CAT*	F-AGCAGGCGGAGAAGTACCCA R-GCTGCTCCACCTCAGCGAAA	XM_026919141.2
*GPx*	F- GTCACTGCAGGATGCAACAC R- TTGGAATTCCGCTCATTGAT	XM_026947312.2
*HSP 70*	F- CTCCTCCTAAACCCCGAGTC R- CCACCAGCACGTTAAACACA	XM_026934573.2
*iNOS*	F-ACACCACGGAGTGTGTTCGT R-GGATGCATGGGACGTTGCTG	XM_026931613.2
*DNA Damage*	F-CACCTTCGCCCTCGAAGTCT R-GCTCGGGTGAGGTCTCTCAG	XM_026938137.2
*TNFα*	F-TGGAGTTCTGCTTGCCGTGG R-GCAGCCTTTGCAGTCTCGGA	XM_026942329.2
*TLR*	F: TCACCACGAACGAGACTTCATCC R : GACAGCACGAAGACACAGCATC	XM_026916808.2
*Ghr1*	FTATTGGCTACAGCTCGCCGC R-AATCACCCCGACTGTGCTGC	XM_034306157.1
*Ghrb*	F-TTGAGCTTTGGGACTCGGAC R-CGTCGATCTTCTCGGTGAGG	XM_026942987.2
*IL1b*	F- AGCAGGATCCATCAAAGTGG R- GTGCTCCAGCTCTCTGGGTA	XM_026918084.2
*Ig*	F- GGCCAGTAATCGTACCTCCA R- CTTCGTAAGGTCCCCACTGA	XM_026923540.2
*MYST*	F-GGGAAAGACCTGGCCGTGAC R-TCGAGGCCGGATTCTCGTCT	XM_026910492.2
*SMT*	F- CTCTGGGTGGCAGAATGAAT R- AACATGAAGAGAACGTTTTCCAG	XM_026921272.2
*GH*	F-CCCAGCAAGAACCTCGGCAA R-GCGGAGCCAGAGAGTCGTTC	GQ859589.1
*CYP P450*	F-GATTCGGCATCCGTGCGTGC R-GATGTGGCTGGGACGAGCA	NC_047599.1
*MT*	F-CACGGCTTTTCCTGTCCGCT R-AACAGCGCCCCCAGGTGTC	AF087935.1
*Cas 3a*	F-CGGCATGAACCAGCGCAAC R-TCCACCGCACCATCTGTCCC	NC_047622.1
*β-Actin*	F-CAGCAAGCAGGAGTACGATG R-TGTGTGGTGTGTGGTTGTTTTG	XM_031749543.1

*SOD* Superoxide dismutase, *CAT* Catalase, *GPx* Glutathione peroxidase, *HSP* Heat shock protein, *iNOS* Nitric oxide synthase, *TNFα* Tumor necrosis factor, *TLR* Toll like receptor, *Ghr* Growth hormone receptor, *IL* Interleukin, *Ig* Immunoglobulin, *MYST* myostatin, *SMT* Somatostatin, *CYP P450* Cytochrome P450, *MT* Metallothionine, *Cas 3a* caspase 3a, *GH* Growth hormone.

### Oxidative stress enzyme activities

Misra and Fridovich^43^ method determined the superoxide dismutase (EC 1.15.1.1). Takahara et al.^44^, Habing et al.^45^, and Paglia and Valentine^46^ were used to determine the catalase (EC 1.11.1.6), glutathione-s-transferase, GST (EC 2.5.1.18) and glutathione peroxidase, GPx (EC 1.11.1.9) respectively. SOD, CAT, GST and GPx were determined in liver, gill and kidney tissues.

### Lipid peroxidation (LPO), Neurotransmitter enzyme and Vitamin C

Uchiyama and Mihara^47^ method were used to determine the LPO in liver, gill and kidney tissues. Hestrin and modified by Augustinsson^48^ method was used to determine the AChE activity in brain tissue. Roe and Keuther^49^ method were used to determine the ascorbic acid in muscle and brain tissues.

### Cortisol

ELISA kit was used to determine the serum cortisol level (Cortisol EIA kit, Catalog no. 500360, Cayman Chemicals, USA) in the serum sample.

### Nitroblue tetrazolium (NBT), serum protein and A:G ratio

Secombes^50^ and modified by Stasiack and Baumann^51^ method used for NBT activity. The serum protein was estimated by using a protein estimation kit (Erba Total Protein Kit, Code no. 120231). Albumin was estimated by Doumas et al.^52^, and globulin was quantified by subtracting albumin values from total plasma protein^53^. The blood glucose was determined as per Nelson^54^ and Somoyogi^55^. The final reading was obtained at 540 nm against the blank.

### Myeloperoxidase content (MPO)

Quade and Roth^56^ with some modifications by Sahu et al.^57^ were used for MPO.

### Metabolic enzymes

Lactate dehydrogenase (LDH; L-lactate NAD1 oxidoreductase; EC.1.1.1.27) was assayed using 0.1 M phosphate buffer (pH 7.5) and 0.2 mM NADH solution in 0.1 M phosphate buffer. The reaction was initiated with addition of substrate 0.2 mM sodium pyruvate and absorbance was recorded at 340 nm^58^ (Wroblewski and Ladue, 1955). A similar reaction mixture was used for the estimation of malate dehydrogenase (MDH; L-malate: NAD^+^ oxidoreductase: EC.1.1.1.37) except for the substrate (1 mg oxaloacetate/ml of chilled triple distilled water)^59^. The activities of aspartate aminotransaminase (AST; EC.2.6.1.1) and alanine amino transaminase (ALT; EC.2.6.1.2) were measured with the help of using oxaloacetate and pyruvate released with the method of Wootten^60^. The LDH, MDH, ALT and AST activities were determined in the liver and kidney tissues.

### Alkaline single-cell gel electrophoresis (SCGE)/comet assay

The DNA damage in gill (50 mg) was determined by alkaline single-cell gel electrophoresis and/or comet assay in gill tissues by three layers of agarose^61^ with slight modification^38^. The tissues were cleaned in double distilled water and chilled phosphate buffer saline (Ca_2_^+^ Mg_2_^+^ free). The tissues were cut into small pieces and homogenized to obtain the single-cell suspension. Then centrifuged at 3000 rpm at 4 °C for 5 min to get cell pellet. The slides were coated with 1% normal agarose (200 µL), mixed with 15 µL of cell suspension (approximately 20 000 cells) with 85 µL of 0.5% low melting point agarose and covered with coverslip. However, after removing the coverslips, the slides were again coated with 100 µL low melting-point agarose. After that, the slides were kept in lysing solution overnight at 4 °C (100 mM Na_2_EDTA, 2.5 M NaCl, 10 mM Tris pH 10 with 10% DMSO and 1% Triton X-100 added fresh). Then slides were placed in gel electrophoresis unit (horizontal) in electrophoresis buffer (1 Mm Na_2_EDTA, 300 mM NaOH, and 0.2% DMSO, pH > 13.5) and the electrophoresis unit for 20 min at 4 °C using 15 V (0.8 V cm1) and 300 mA. Then slides were washed 3 times in neutralizing buffer with 0.4 M tris buffer (pH 7.5). The slides were stained with 75 µL ethidium bromide (20 µg ml^-1^) for 5 min to visualize DNA damage. Then slides were analysed in a fluorescent microscope (Leica Microsystems Ltd, DM 2000, Heerbrugg, Switzerland), captured photographs, and analysed in an image analysis system with Open comet.

### Growth performance

The growth performance of the fish was determined as per our previous method^36^. The growth performance attributes such as feed conversion ratio (FCR), Weight gain (%), protein efficiency ratio (PER), specific growth rate, thermal growth coefficient (TGC), relative feed intake (RFI), and daily growth index (DGI), were determined in the study. The weight of the fish was observed every 15 days, up to 105 days.

$$\begin{aligned} {\text{FCR}} & = {\text{Total}}\;{\text{ dry}}\;{\text{ feed}}\;{\text{ intake}}\left( {\text{g}} \right)/{\text{Wet}}\;{\text{weight}}\;{\text{gain }}\left( {\text{g}} \right) \\ {\text{SGR}} & = {1}00\left( {{\text{ln FBW}} - {\text{ln IBW}}} \right)/{\text{number of days}} \\ {\text{Weight}}\;{\text{gain}}\left( \% \right) & = {\text{Final}}\;{\text{ body}}\;{\text{ weight}}\left( {{\text{FBW}}} \right) - {\text{Initial}}\;{\text{ body }}\;{\text{weight}}\left( {{\text{IBW}}} \right)/{\text{Initial}}\;{\text{ body }}\;{\text{weight }}\left( {{\text{IBW}}} \right) \times {1}00 \\ {\text{Relative}}\;{\text{feed}}\;{\text{intake}},\left( {{\text{FI}}} \right)\left( {\% /{\text{d}}} \right) & = {1}00 \times \left( {{\text{TFI}}/{\rm I}{\text{BW}}} \right) \\ {\text{PER}} & = {\text{Total}}\;{\text{ wet}}\;{\text{ weight}}\;{\text{ gain}}\left( {\text{g}} \right)/{\text{crude}}\;{\text{ protein}}\;{\text{intake }}\left( {\text{g}} \right) \\ {\text{Thermal}}\;{\text{growth}}\;{\text{coefficient}},\left( {{\text{TGC}}} \right) & = \left( {{\text{FBW}}^{{{1}/{3}}} {-}{\text{IBW}}^{{{1}/{3}}} } \right) \times \left( {\Sigma {\text{D}}0} \right)^{{ - {1}}} ,{\text{where }}\;\Sigma {\text{D}}0\;{\text{ is}}\;{\text{ the}}\;{\text{ thermal }}\;{\text{sum }}\;\left( {{\text{feeding}}\;{\text{ days }} \times {\text{ average}}\;{\text{ temperature}},^\circ {\text{C}}} \right) \\ {\text{Daily}}\;{\text{ growth}}\;{\text{ index}},{\text{DGI }}\left( \% \right) & = \left( {{\text{FBW}}^{{{1}/{3}}} {-}{\text{ IBW}}^{{{1}/{3}}} } \right)/{\text{days}} \times {1}00 \\ \end{aligned}$$

### Sample preparation for analysis of arsenic and copper

Liver, muscle, gill, brain, and kidney were collected to determine arsenic concentration. The tissues and diets were processed in a microwave digestion system (Microwave Reaction System, Multiwave PRO, Anton Paar GmbH, Austria, Europe) using Inductively Coupled Plasma Mass Spectrometry (ICP-MS) (Agilent 7700 series, Agilent Technologies, USA) as followed the method of Kumar et al.^62,63^.

### Challenge study with *Aeromonas hydrophila*

*Aeromonas hydrophilla* (Lot no. 637-51-5 and Ref 0637P, HiMedia, Mumbai) was injected into *P. hypophthalmus* at the end of the 105 days experimental trial. The bacteria were cultured in nutrient broth at 37 °C for 24 h in an orbital shaker, and then the culture was harvested using centrifugation at 6000 rpm at 4 °C for 15 min. The culture was washed in PBS (pH 7.2) and maintained the10^8^ CFU mL^-1^ count. The suspension of 0.15 mL was injected into fish and observed the mortality for a week.

$$\begin{aligned} {\text{Relative}}\% {\text{ survival }} & = \frac{{{\text{Mortality }}\left( \% \right){\text{ Control}}{-}{\text{Mortality }}\left( \% \right){\text{Treatment}}}}{{{\text{Mortality}}\left( \% \right){\text{Control}}}} \, \times {1}00 \\ {\text{Cumulative}}\;{\text{ mortality}}\left( \% \right) & = \frac{{{\text{Total}}\;{\text{ mortality}}\;{\text{ in }}\;{\text{each }}\;{\text{treatment}}\;{\text{ after}}\;{\text{ challenge}}}}{{{\text{Total}}\;{\text{ no}}. \, \;{\text{of }}\;{\text{fish }}\;{\text{challenged}}\;{\text{for }}\;{\text{the}}\;{\text{ same}}\;{\text{ treatments}}}} \times {1}00. \\ \end{aligned}$$

### Statistical analysis

The Statistical Package for Social Sciences program (SPSS 16) was used for data analysis. The data were tested for homogeneity and normality of variance using Levene's and Shapiro–Wilk’s test, respectively. If both tests were satisfied, one-way Analysis of variance (ANOVA) with DMRT (Duncan's multiple range tests) was employed to test the statistically significant difference at p < 0.05. The data were expressed as mean ± SE.

### Consent to participate

All authors are aware and agree with this submission for publication.

## Results

### Primary stress response elevated by non-lethal dose of arsenic, low pH (6.5) and high temperature (34 °C) but dietary copper mitigate it

**Figure 1:**
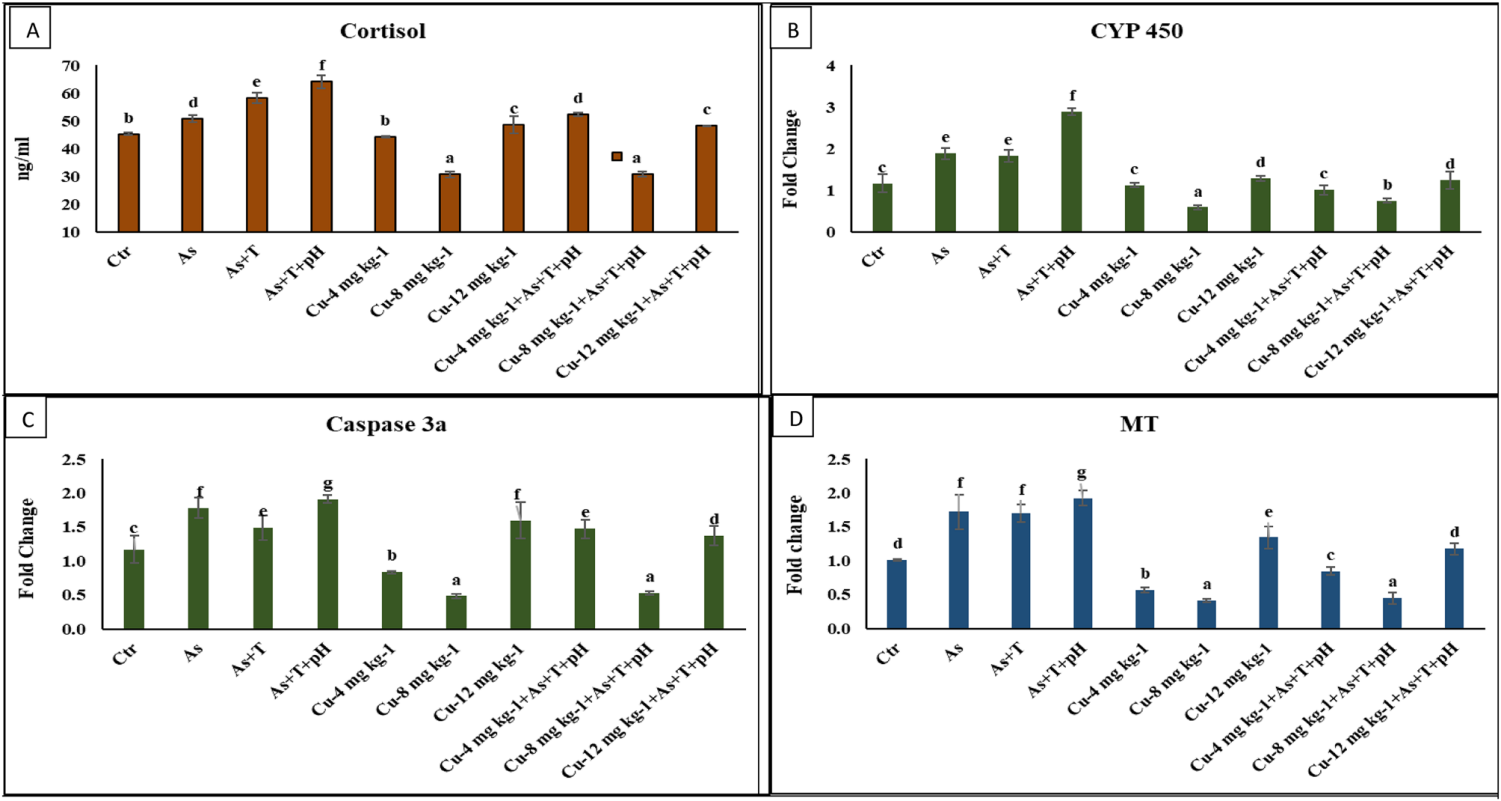
(**A**–**D**) Effect of dietary copper (Cu) to improve cortisol and gene expression of CYP 450, Caspase 3a, metallothionine (MT) in *P. hypophthalmus* reared under control or arsenic, low pH (6.5) and high temperature stress (34 °C) for 105 days. Within endpoints and groups, bars with different superscripts differ significantly (**a**–**d**). Data expressed as Mean ± SE (n = 3).

A non-lethal dose of arsenic, low pH, and high temperature (As + pH + T) stress was significantly elevated (*p* = 0.0039) the serum cortisol levels followed by arsenic and high temperature (As + T) and arsenic alone group compared to control and dietary copper groups. Dietary Cu at 8 mg kg^-1^ diet fed group noticeably reduced the cortisol level compared to control and other groups in *P. hypophthalmus* (Fig. 1A).

### Secondary stress response (*CYP 450, CAS 3a* and *MT*) elevated by non-lethal dose of arsenic, low pH (6.5) and high temperature (34 °C), but dietary copper mitigate it

Cytochrome P450 (*CYP 450*) (*p* = 0.0021) and metallothionine (*MT*) (*p* = 0.001) genes regulation in liver were noticeably upregulated with concurrent exposure to low dose of arsenic, low pH and high-temperature stress (As + pH + T) followed by As + T and As exposure group in compared to control and dietary Cu supplemented groups. Whereas, caspase 3a (*CAS 3a*) gene regulation was significantly (*p* = 0.0011) upregulated with exposure to As + pH + T followed by As alone and As + T group and fed with control diet compared to control and Cu supplemented diet. Indeed, the Cu dietary group at 8 mg kg^-1^ diet with or without stressors was remarkably downregulated in the *CYP 450*, *MT*, and *Cas 3a* gene regulation compared to control and stressors groups. In the case of *CYP 450*, the Cu at 4 mg kg^-1^ diet in stressors and non-stressors groups was similar to the control group, whereas the Cu at 12 mg kg^-1^ diet in the *CYP 450* was highly upregulated to the control group. Similarly, the gene regulation of *Cas 3a* in a group fed with Cu at 4 mg kg^-1^ diet without stressors was significantly lowered. In contrast, Cu at 4 mg kg^-1^ diet with stressors was considerably higher compared to the control group. The group fed with Cu at 12 mg kg^-1^ diet (without stressors) and Cu at 4 and 12 mg kg^-1^ diet with stressors showed higher gene regulation than the control group. The results of *CYP 450*, *Cas 3a*, and *MT* genes were effectively modulating the gene expressions against multiple stressors (As + pH + T) (Fig. 1B–D).

### Secondary stress response (*HSP 70, iNOS* and DNA damage) elevated by non-lethal dose of arsenic, low pH (6.5) and high temperature (34 °C) but dietary copper mitigate it

**Figure 2:**
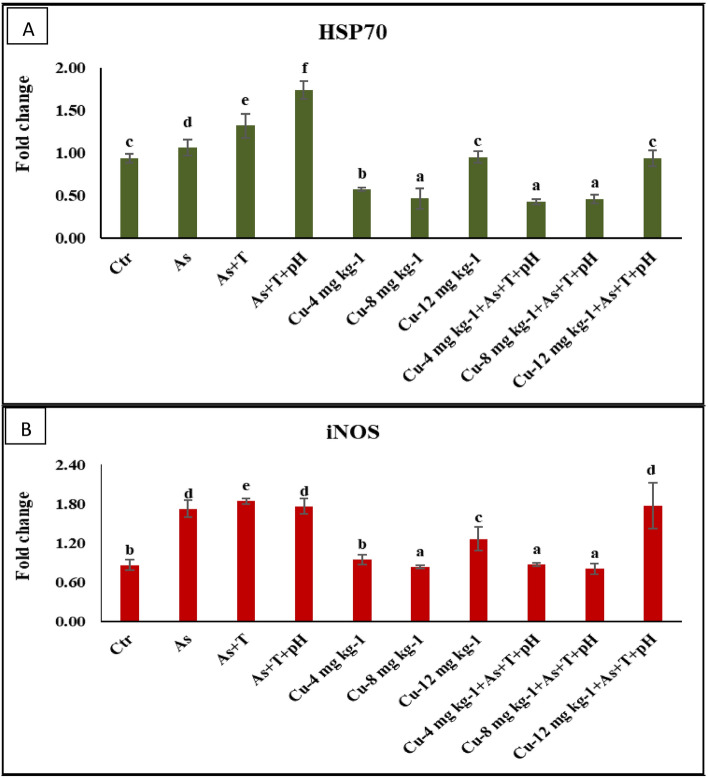
(**A**, **B**) Effect of dietary copper (Cu) on gene expression of HSP 70, and iNOS in *P. hypophthalmus* reared under control or arsenic, low pH (6.5) and high temperature stress (34 °C) for 105 days. Within endpoints and groups, bars with different superscripts differ significantly (**a**–**d**). Data expressed as Mean ± SE (n = 3).

The gene expression of *HSP 70* in the liver was remarkably upregulated (*p* = 0.0018) with concurrent exposure to a low dose of arsenic, low pH, and high temperature followed by arsenic and temperature as well as As alone exposure group in comparison to control and Cu supplemented groups. Moreover, *HSP 70* gene was downregulated in the group fed with Cu at 4 and 8 mg kg^-1^ diet with or without stressors compared to control and stressors groups (Fig. 2A). Further, *iNOS* gene expression was noticeably upregulated (*p* = 0.0007) in group exposed to arsenic and temperature followed by As + pH + T and As alone group and fed with a control diet compared to control and Cu supplemented groups. Whereas, dietary Cu at 8 mg kg^-1^ diet with or without stressors groups and Cu at 4 mg kg^-1^ diet with stressors significantly downregulated the *iNOS* gene expression compared to control and other groups (Fig. 2B). In the present investigation, the DNA damage was also determined using single cell gel electrophoresis/comet assay to be considered as comet area, comet length, comet DNA, head area, head DNA, head DNA (%), tail area, tail DNA and tail DNA (%). The highest tail DNA (%) was determined in the group concurrent exposed to As + pH + T (95%), followed by As + T (87%) and As (81%). Whereas, the tail DNA (%) in the group treated with control and Cu at 4, 8, and 12 mg kg^-1^ diet without stressors were 16, 16, 11, and 27%, respectively, and in the case of stressor group fed with Cu at 4, 8 and 12 mg kg-1 diet were 20, 18 and 43% (Table 4).

**Table 4:** Dietary copper protects the tissue against DNA damage in *P. hypophthalmus* reared under control or arsenic, low pH (6.5) and high temperature stress (34 °C) stress for 105 days.

Treatments	Non-Stressors	Stressors			Non-Stressors			Stressors (As + T + pH)		
		As	As + T	As + T + pH	Cu-4.0 mg kg^-1^	Cu-8.0 mg kg^-1^	Cu-12.0 mg kg^-1^	Cu-4.0 mg kg^-1^	Cu-8.0 mg kg^-1^	Cu-12.0 mg kg^-1^
Diet	Control	Control	Control	Control						
Comet Area	719 ± 3.71	649 ± 3.81	794 ± 6.68	1610 ± 3.98	719 ± 3.27	868 ± 11.72	520 ± 4.98	448 ± 6.37	621 ± 8.15	774.2 ± 5.38
Comet Length	22 ± 1.03	23 ± 1.38	31 ± 1.27	49 ± 1.97	22 ± 1.22	25 ± 1.28	18 ± 1.35	16 ± 1.57	25 ± 1.56	28 ± 1.12
Comet DNA	8972 ± 12.9	15,524 ± 11.79	136,025 ± 13.8	290,280 ± 4.28	8972 ± 11.56	175,464 ± 34.2	56,925 ± 54.7	93,179 ± 54.3	85,860 ± 34.9	104,686 ± 34.9
Head Area	600 ± 3.98	80 ± 2.91	108 ± 1.87	52 ± 1.98	600 ± 5.57	699 ± 4.91	394 ± 6.38	359 ± 3.78	528 ± 8.46	312 ± 2.98
Head DNA	7502 ± 1.98	2883 ± 11.64	16,634 ± 13.76	12,732 ± 12.98	7502 ± 14.98	155,944 ± 24.3	41,122 ± 23.8	74,359 ± 65.4	69,937 ± 42.92	37,643 ± 7.35
Head DNA (%)	83.62 ± 2.67	18.57 ± 1.18	12.23 ± 1.97	4.39 ± 0.78	83.62 ± 4.28	88.88 ± 5.34	72.24 ± 5.37	79.80 ± 2.67	81.45 ± 4.34	56.63 ± 3.28
Tail Area	119 ± 5.28	569 ± 12.98	686 ± 2.58	1558 ± 11.93	119 ± 4.26	169 ± 1.87	126 ± 2.71	89 ± 2.79	93 ± 3.28	462.2 ± 2.98
Tail DNA	1470 ± 12.87	12,641 ± 15.89	119,391 ± 13.6	277,548 ± 23.6	1470 ± 3.37	19,520 ± 25.7	15,803 ± 26.3	18,820 ± 11.45	15,923 ± 12.9	67,042 ± 3.11
Tail DNA (%)	16.38 ± 1.68	81.43 ± 2.67	87.77 ± 3.27	95.61 ± 2.56	16.38 ± 1.63	11.12 ± 1.01	27.76 ± 2.18	20.20 ± 1.17	18.55 ± 1.67	43.37 ± 2.98

Data expressed as Mean ± SE (n = 3).

### Secondary stress response (SOD, CAT, GST, GPx, and LPO) elevated by non-lethal dose of arsenic, low pH (6.5) and high temperature (34 °C) but dietary copper mitigate it

**Figure 3:**
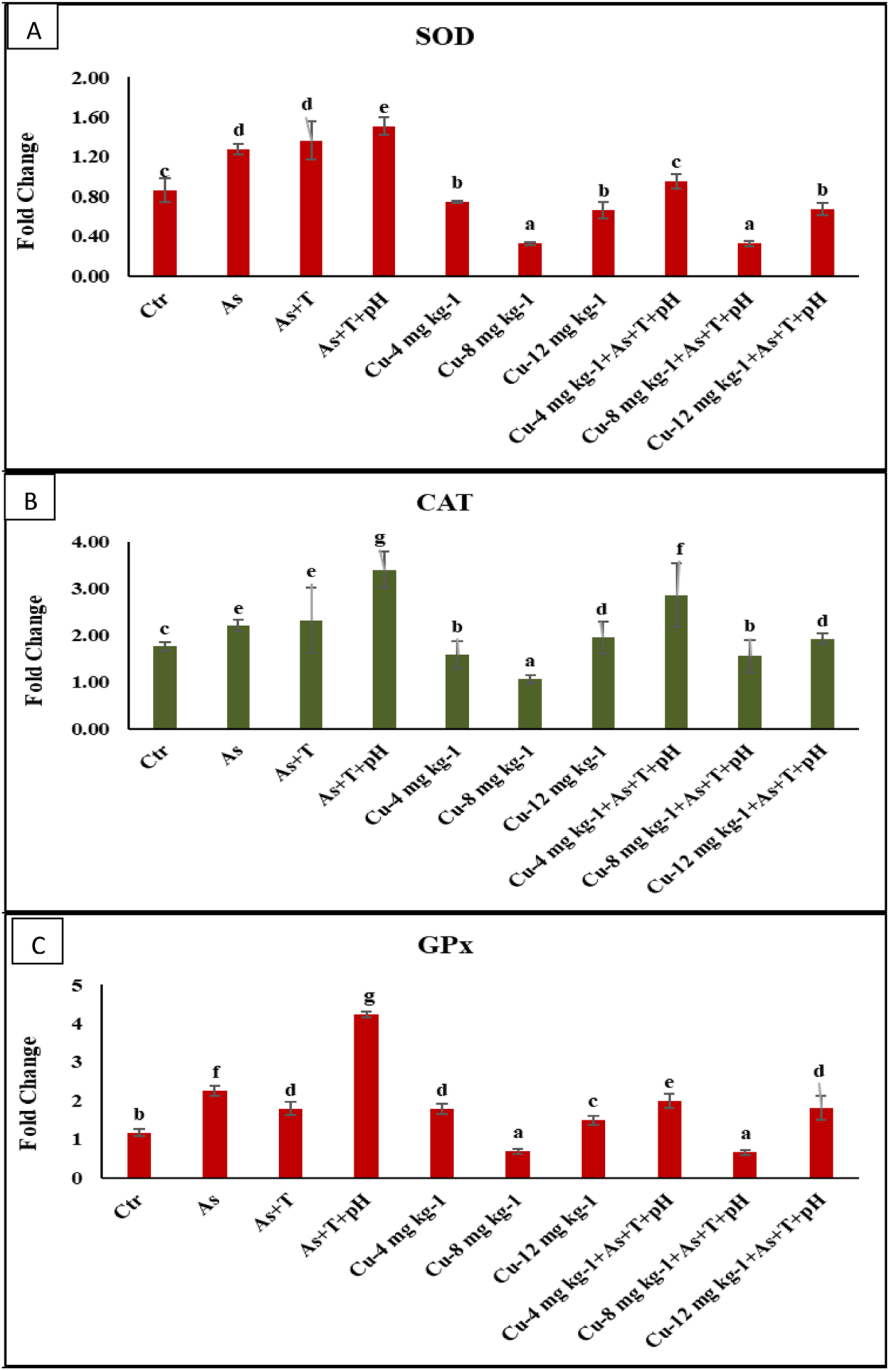
(**A**–**C**): Effect of dietary copper (Cu) on gene expression of CAT, SOD and GST in *P. hypophthalmus* reared under control or arsenic, low pH (6.5) and high temperature stress (34 °C) for 105 days. Within endpoints and groups, bars with different superscripts differ significantly (**a**–**d**). Data expressed as Mean ± SE (n = 3).

*SOD, CAT,* and *GPx* gene expression in liver tissue as well as biochemical analysis were performed in the present investigation. The gene expression of *SOD* was significantly upregulated (*p* = 0.0012) with concurrent exposure to a low dose of arsenic, low pH, and high temperature, followed by the As + T and As a group compared to control and other groups. Similarly, *CAT* gene regulation was significantly upregulated (*p* = 0.0017) with As + pH + T group followed by Cu at 4 mg kg^-1^ diet with stressors and As + T and As group compared to control and other groups. Whereas, in case of gene expression *GPx*, a similar pattern was obtained as the *CAT* gene in *P. hypophthalmus*. Indeed, the *SOD, CAT*, and *GPx* gene expressions were significantly downregulated with dietary Cu at 8 mg kg^-1^ diet compared to control and other groups (Fig. 3A–C).

The enzymatic activities of SOD, CAT, GPx, and GST in gill, liver, and kidney tissues of *P. hypophthalmus* were determined and presented in Table 5. CAT activities in liver (*p* = 0.0063) and kidney (*p* = 0.0023) tissues were noticeably elevated with concurrent exposure to a low dose of arsenic, low pH, and high temperature, followed by As + T and As exposure group compared to control and other groups. Whereas CAT activity in the liver was significantly (*p* = 0.0029) higher with exposure to As + T and As + pH + T followed by the As alone group. Further, the CAT activities in liver, kidney and gill tissues were noticeably reduced with dietary Cu at 8 mg kg^-1^ diet with or without stressors compared to control and other groups. Except liver tissues, dietary Cu at 4 and 12 mg kg^-1^ diet did not reduce the CAT activities compared to control and other groups. Exposure to a low dose of arsenic, low pH, and high temperature (As + pH + T) and As + T significantly elevated the SOD activities in the liver (*p* = 0.035), and gill (*p* = 0.022) tissues in comparison to control and Cu diets supplemented groups. Whereas, in the case of kidney SOD, the activity was significantly elevated (*p* = 0.019) with exposure to As + pH + T followed by As + T and As group. Dietary Cu at 8 mg kg^-1^ diet group in stressors and without stressors significantly depresses the SOD activities in all the tissues compared to control and other groups. Moreover, in case of GST activities in liver (*p* = 0.0031), kidney (*p* = 0.0058) and gill (*p* = 0.0045) were noticeably elevated with concurrent exposure to low dose of arsenic, low pH and high temperature followed by group exposed to arsenic and high temperature and arsenic alone group compared to control and Cu supplemented groups. GST and GPx activities in all the tissues were significantly reduced with supplementation of dietary Cu at 8 mg kg^-1^ diet with or without stressors compared to control and other groups. Further, the GPx activities in the liver (*p* = 0.0076), kidney (*p* = 0.0037), and gill (p = 0.0014) were noticeably enhanced with the As + pH + T group, followed by As + T and As group. The lipid peroxidation (LPO) in the gill (*p* = 0.013), liver (*p* = 0.0027), and kidney (*p* = 0.018) were significantly elevated with exposure to the As + pH + T group, followed by As + T and As groups compared to control and Cu supplemented groups. Further, LPO was lowered considerably in the Cu-fed group at 8 mg kg^-1^ diet with or without stressors. The other Cu-supplemented groups were similar functions to the control diet group (Table 6).

**Table 5:** Effect of dietary copper (Cu) to improve the CAT, SOD, GST and GPx activities in *P. hypophthalmus* reared under control or arsenic, low pH (6.5) and high temperature stress (34 °C) for 105 days.

Treatments	Non-Stressors	Stressors			Non-Stressors			Stressors (As + T + pH)			*p*-value
		As	As + T	As + T + pH	Cu-4.0 mg kg^-1^	Cu-8.0 mg kg^-1^	Cu-12.0 mg kg^-1^	Cu-4.0 mg kg^-1^	Cu-8.0 mg kg^-1^	Cu-12.0 mg kg^-1^	
Diet	Control	Control	Control	Control							
CAT-L	11.94^c^ ± 0.45	13.53d ± 0.65	17.76e ± 1.37	20.19f. ± 1.17	9.14b ± 1.08	5.13a ± 0.99	11.89c ± 1.43	10.43c ± 1.11	5.74a ± 0.91	11.69c ± 0.89	0.0063
CAT-G	7.55^b^ ± 0.85	13.86d ± 0.90	17.56e ± 1.21	16.45e ± 1.30	7.94b ± 0.62	4.43a ± 0.64	9.97c ± 1.26	7.94b ± 0.99	5.02a ± 0.97	10.18c ± 0.85	0.0029
CAT-K	10.70^b^ ± 0.70	14.78d ± 1.66	19.74e ± 0.55	22.68f. ± 0.88	11.30b ± 0.65	6.51a ± 1.04	11.11b ± 1.26	10.26b ± 0.89	6.88a ± 1.13	13.12c ± 0.88	0.0023
SOD-L	56.47^b^ ± 0.55	62.70d ± 1.26	63.43de ± 1.44	64.99e ± 0.38	58.06bc ± 2.08	52.46a ± 0.40	60.23c ± 1.22	60.31c ± 1.85	52.80a ± 0.59	62.0d ± 2.17	0.035
SOD-G	39.99c ± 1.38	43.04d ± 0.64	44.47de ± 0.39	45.41e ± 0.61	37.86b ± 1.19	36.43a ± 0.64	40.84c ± 1.30	37.93b ± 1.90	36.82a ± 0.37	39.93c ± 1.22	0.022
SOD-K	66.28c ± 0.27	68.99d ± 1.77	69.96d ± 0.63	71.41e ± 1.93	65.52c ± 1.19	60.42a ± 0.61	68.93d ± 1.34	69.02d ± 1.97	62.09b ± 0.86	69.48d ± 1.40	0,019
GST-L	0.30b ± 0.03	0.53c ± 0.02	0.58d ± 0.01	0.70e ± 0.03	0.30b ± 0.02	0.19a ± 0.02	0.32b ± 0.02	0.30b ± 0.03	0.18a ± 0.02	0.33b ± 0.03	0.0031
GST-G	0.41b ± 0.05	0.80c ± 0.04	0.89d ± 0.10	1.08e ± 0.10	0.44b ± 0.02	0.24a ± 0.04	0.48b ± 0.08	0.49b ± 0.02	0.25a ± 0.03	0.47b ± 0.02	0.0045
GST-K	0.56c ± 0.03	0.79d ± 0.03	0.86e ± 0.06	0.97f. ± 0.02	0.60c ± 0.02	0.28a ± 0.03	0.50b ± 0.07	0.50b ± 0.03	0.31a ± 0.03	0.51b ± 0.03	0.0058
GPx-L	4.22b ± 0.44	6.34c ± 0.45	8.21d ± 0.81	9.90e ± 0.71	4.84b ± 0.58	2.70a ± 0.32	4.36b ± 0.41	4.94b ± 0.14	2.00a ± 0.13	4.88b ± 0.53	0.0076
GPx-G	0.49b ± 0.04	0.75c ± 0.20	0.76c ± 0.12	1.24d ± 0.12	0.50b ± 0.05	0.32a ± 0.03	0.52b ± 0.08	0.58b ± 0.06	0.38a ± 0.02	0.49b ± 0.06	0.014
GPx-K	0.56b ± 0.07	0.87c ± 0.05	0.92c ± 0.07	1.06d ± 0.03	0.60b ± 0.09	0.34a ± 0.02	0.62b ± 0.06	0.58b ± 0.09	0.35a ± 0.04	0.58b ± 0.09	0.0037

Values in the same row with different superscript (a, b, c, d, e, f) differ significantly. Data expressed as Mean ± SE (n = 6). Catalase, SOD, GST and GPx: Units/mg protein.

**Table 6:** Effect of dietary copper (Cu) to improve the LPO, LDH, MDH, ALT, AST, Vit C and AChE in *P. hypophthalmus* reared under control or arsenic, low pH (6.5) and high temperature stress (34 °C) for 105 days.

Treatments	Non-Stressors	Stressors			Non-Stressors			Stressors (As + T + pH)			*p*-value
		As	As + T	As + T + pH	Cu-4.0 mg kg^-1^	Cu-8.0 mg kg^-1^	Cu-12.0 mg kg^-1^	Cu-4.0 mg kg^-1^	Cu-8.0 mg kg^-1^	Cu-12.0 mg kg^-1^	
Diet	Control	Control	Control	Control							
LPO-L	16.33b ± 0.51	22.82d ± 0.37	26.17e ± 0.50	29.77f. ± 0.45	16.0b ± 0.50	12.10a ± 0.91	18.01c ± 1.32	17.42bc ± 0.58	13.12a ± 0.55	16.61b ± 0.85	0.013
LPO-G	12.09b ± 0.44	17.76d ± 0.72	21.42e ± 0.38	23.50f. ± 0.79	11.28b ± 0.40	8.91a ± 0.32	17.15d ± 0.60	12.02b ± 0.89	8.72a ± 0.52	14.35c ± 0.26	0.0027
LPO-K	18.20b ± 0.53	24.49c ± 0.40	26.92d ± 0.45	25.73d ± 0.44	18.63b ± 0.40	12.38a ± 0.38	20.99bc ± 0.52	21.72bc ± 1.14	11.81a ± 0.53	18.37b ± 0.28	0.018
LDH-L	0.43bc ± 0.04	0.56d ± 0.02	0.75e ± 0.05	0.97f. ± 0.02	0.39b ± 0.06	0.24a ± 0.02	0.50c ± 0.04	0.55d ± 0.04	0.25a ± 0.03	0.56d ± 0.02	0.012
LDH-K	0.64c ± 0.05	0.91d ± 0.03	1.31e ± 0.05	1.24e ± 0.03	0.54b ± 0.04	0.29a ± 0.02	0.57b ± 0.04	0.52b ± 0.03	0.28a ± 0.02	0.49b ± 0.09	0.039
MDH-L	1.32c ± 0.13	3.24e ± 0.25	4.20f. ± 0.23	4.82 g ± 0.33	1.02b ± 0.08	0.68a ± 0.07	2.21d ± 0.21	0.97b ± 0.04	0.70a ± 0.04	1.10b ± 0.06	0.027
MDH-K	1.69d ± 0.13	3.52e ± 0.41	4.36f. ± 0.28	5.42 g ± 0.35	0.95c ± 0.16	0.62a ± 0.07	1.10c ± 0.04	0.95c ± 0.07	0.74b ± 0.06	1.13c ± 0.11	0.017
ALT-L	21.18c ± 2.07	27.36 ± 2.13	35.59 ± 2.84	41.61 ± 2.07	18.50b ± 1.77	13.63a ± 1.12	23.20d ± 2.82	22.60c ± 0.82	14.77a ± 0.84	23.38d ± 0.90	0.0016
ALT-K	9.79c ± 0.56	14.96d ± 0.86	19.90e ± 1.44	22.46f. ± 0.95	7.55b ± 1.54	2.70a ± 0.30	7.96b ± 0.73	6.99b ± 0.46	2.38a ± 0.81	9.11c ± 084	0.0028
AST-L	60.28d ± 1.55	65.79e ± 1.60	65.96e ± 2.09	77.14f. ± 2.24	53.74c ± 2.15	45.11b ± 2.48	55.24d ± 1.37	51.04c ± 1.22	42.13a ± 2.33	52.41c ± 1.82	0.0043
AST-K	12.91d ± 0.89	19.00e ± 1.45	22.46f. ± 1.55	26.36 g ± 0.57	13.08d ± 0.70	3.97a ± 0.60	9.46c ± 0.80	6.65b ± 1.01	4.10a ± 0.54	9.90c ± 0.49	0.0055
Vit C-M	19.94d ± 0.46	16.49c ± 0.66	13.31b ± 0.37	10.09a ± 0.20	22.40e ± 0.33	27.73f. ± 0.63	18.47d ± 0.75	20.69d ± 0.31	32.42 g ± 0.87	19.70d ± 0.34	0.0043
Vit C-B	20.22d ± 0.41	16.54c ± 0.27	12.94b ± 0.45	9.55a ± 0.31	23.44e ± 0.34	29.21f. ± 0.59	19.66d ± 0.56	21.05d ± 0.42	29.58f. ± 0.64	19.21d ± 0.69	0.0066
AChE-B	0.47d ± 0.02	0.38c ± 0.01	0.29b ± 0.01	0.23a ± 0.02	0.52e ± 0.04	0.63f. ± 0.05	0.35c ± 0.01	0.52e ± 0.03	0.60f. ± 0.04	0.33c ± 0.02	0.0017

Values in the same row with different superscript (a, b, c, d, e) differ significantly. Data expressed as Mean ± SE (n = 6). LPO: n mole TBARS formed/h/mg protein; LDH and MDH: units/min/mg protein at 37 °C; ALT: nmole of sodium pyruvate formed/ mg protein/min at 37 °C; AST: nmole Oxaloacetate released/min/mgproteinat37 °C; AChE: nmole/min/mg protein; Vit C:µg/g of wet tissue.

### Secondary stress response (LDH, MDH, ALT, AST, Vit C, and AChE) elevated by non-lethal dose of arsenic, low pH (6.5) and high temperature (34 °C) but dietary copper mitigate it

Table 6 summarises the results of LDH, MDH, ALT, AST activities in liver and kidney, and Vit C in muscle and brain in *P. hypophthalmus*. Concurrent exposure to low dose of arsenic, low pH, and the high temperature noticeably enhances (*p* < 0.05) the LDH, MDH, ALT, and AST activities in liver and kidney tissues compared to control and Cu supplemented diet groups. In case of LDH and ALT in liver and kidney as well as MDH in liver and AST in kidney were remarkably reduced (*p* < 0.05) with dietary Cu at 8 mg kg^-1^ diet fed group with or without stressors compared to control and other groups followed by Cu at 4 mg kg^-1^ diet group. In contrast to the above results, the MDH activities in the kidney and AST activities in the liver were significantly lowered with supplementation of Cu at 8 mg kg^-1^ diet with the stressors group and vice versa. The Cu at 4 mg kg^-1^ diet with or without stressors and 12 mg kg^-1^ without stressors effectively modulate the As + pH + T stressor in *P. hypophthalmus*. Vit C in muscle (*p* = 0.0045) and brain (*p* = 0.0066) tissues were significantly elevated with supplementation of dietary Cu at 8 mg kg^-1^ diet with or without stressors followed by Cu at 4 mg kg^-1^ diet without stressors to control and other groups. However, Vit C was significantly reduced in group treated under As + pH + T followed by As + T and As group. Similarly, AChE activities were noticeably inhibited with stressors (As, As + T, and As + pH + T), whereas supplementation of Cu diet at 8 mg kg^-1^ diet followed by 4 mg kg^-1^ diet with or without stressors compared to control and other groups (Table 6).

### Secondary stress response (Total protein, albumin, globulin, A:G ratio, NBT and blood glucose) elevated by non-lethal dose of arsenic, low pH (6.5) and high temperature (34 °C) but dietary copper mitigate it

The results revealed that the fish's immunity (Total protein, albumin, globulin, A: G ratio, NBT, and MPO) was noticeably reduced with exposure to stressors As + pH + T, As + T, and As. The total protein (*p* = 0.016), globulin (*p* = 0.032), NBT (*p* = 0.0055), and MPO (*p* = 0.0046) were potentially elevated with dietary Cu at 8 mg kg^-1^ diet with or without stressors compared to control and other groups. TP, globulin, NBT, and MPO were significantly reduced with As + pH + T followed by As + T and As and fed with a control diet compared to control and Cu supplemented groups. In contrast to these results, the A:G ratio (*p* = 0.0018) and blood glucose (*p* = 0.0027) were noticeably reduced with supplementation of the Cu diet at 8 mg kg^-1^ compared to the control and stressors group. In the case of albumin (*p* = 0.025), the Cu supplemented group at 4 and 8 mg kg^-1^ diet was similar to the control group, whereas the Cu diet was effective against stressors (As + pH + T, As + T, and As) groups (Table 7).

**Table 7:** Effect of dietary copper (Cu) to improve the Total protein, albumin, globulin, A:G ratio, nitro blue tetrazolium NBT, blood glucose and MPO in *P. hypophthalmus* reared under control or arsenic, low pH (6.5) and high temperature (34 °C) stress for 105 days.

Treatments	Non-Stressors	Stressors			Non-Stressors			Stressors (As + T + pH)			*p*-Value
		As	As + T	As + T + pH	Cu-4.0 mg kg^-1^	Cu-8.0 mg kg^-1^	Cu-12.0 mg kg^-1^	Cu-4.0 mg kg^-1^	Cu-8.0 mg kg^-1^	Cu-12.0 mg kg^-1^	
Diet	Control	Control	Control	Control							
Total Protein	0.63d ± 0.03	0.56c ± 0.02	0.45b ± 0.02	0.37a ± 0.03	0.57c ± 0.03	0.97e ± 0.03	0.51bc ± 0.03	0.56c ± 0.02	1.02e ± 0.03	0.51bc ± 0.04	0.016
Albumin	0.15a ± 0.01	0.16b ± 0.02	0.20c ± 0.01	0.15a ± 0.01	0.13 ± 0.01	0.14a ± 0.01	0.16b ± 0.02	0.17c ± 0.01	0.11a ± 0.01	0.16b ± 0.01	0.025
Globulin	0.48c ± 0.03	0.40b ± 0.02	0.25a ± 0.02	0.22a ± 0.02	0.44bc ± 0.02	0.83d ± 0.03	0.35b ± 0.02	0.39b ± 0.02	0.91d ± 0.04	0.35b ± 0.05	0.032
A:G ratio	0.32b ± 0.03	0.39c ± 0.06	0.83f. ± 0.06	0.69e ± 0.06	0.29b ± 0.00	0.17a ± 0.02	0.45d ± 0.07	0.44d ± 0.04	0.12a ± 0.01	0.46d ± 0.07	0.0018
NBT	0.58d ± 0.02	0.46c ± 0.04	0.35b ± 0.03	0.26a ± 0.01	0.60d ± 0.03	0.66e ± 0.05	0.50c ± 0.03	0.51c ± 0.03	0.73f. ± 0.02	0.51c ± 0.03	0.0055
Blood Glucose	96.95c ± 2.71	129.34f. ± 2.38	145.96 g ± 5.91	170.36 h ± 4.51	90.26b ± 2.93	70.93a ± 0.98	118.11e ± 4.22	99.97d ± 3.69	72.27a ± 2.2	119.41e ± 2.7	0.0027
MPO	0.44d ± 0.02	0.23b ± 0.01	0.20a ± 0.01	0.17a ± 0.02	0.44d ± 0.02	0.64e ± 0.02	0.35c ± 0.01	0.46d ± 0.03	0.65e ± 0.04	0.36c ± 0.03	0.0046

Values in the same row with different superscript (a, b, c, d, e) differ significantly. Total protein, albumin, globulin: g dL^-1^Blood glucose: mgdL^-1^; Data expressed as Mean ± SE (n = 3).

### Secondary stress response (*TNFα, TLR, *and *Ig*) elevated by non-lethal dose of arsenic, low pH (6.5), and high temperature (34 °C), but dietary copper mitigates it

**Figure 4:**
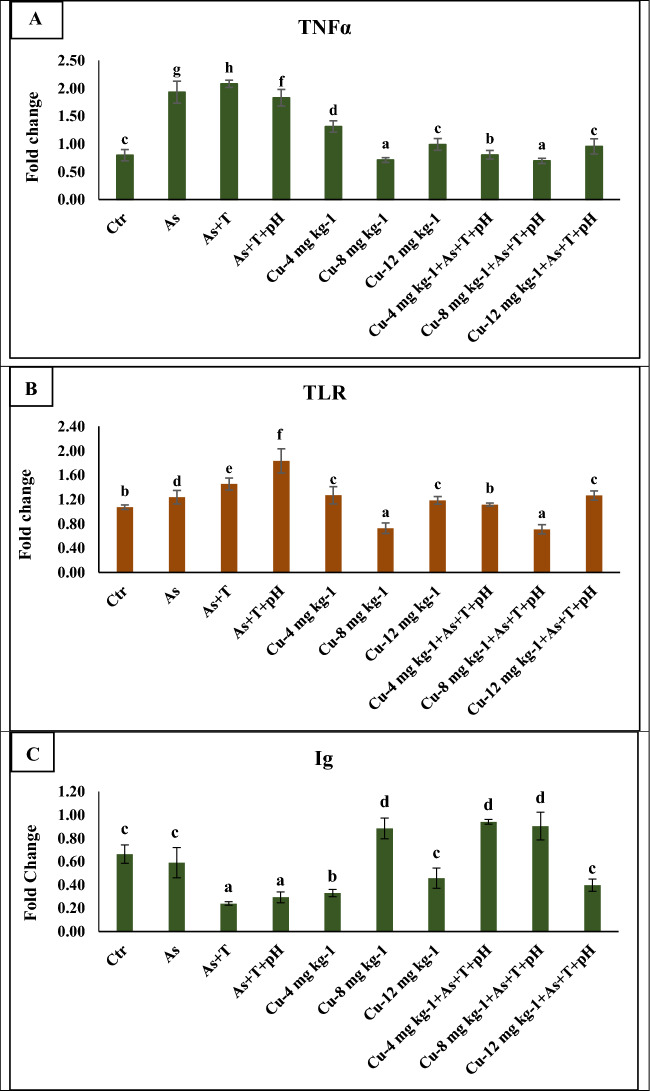
(**A**–**C**): Effect of dietary copper (Cu) on gene expression of TNFα, TLR, and Ig in *P. hypophthalmus* reared under control or arsenic, low pH (6.5) and high temperature stress (34 °C) for 105 days. Within endpoints and groups, bars with different superscripts differ significantly (**a**–**d**). Data expressed as Mean ± SE (n = 3).

The data on *TNFα, TLR,* and *Ig* are recoded in Fig. 4A–C. The important immunological gene regulation in terms of *TNFα, TLR,* and *Ig* were analysed in liver tissues of *P. hypophthalmus*. The results of *TNFα* (*p* = 0.0016), was potentially upregulated with concurrent exposure to low dose of arsenic and high-temperature group followed by As + pH + T and As group, whereas *TLR* (p = 0.0034) was significantly upregulated in the group treated with As + pH + T followed by As + T and As in comparison to control and other groups. Further, *TNFα*, and *TLR* gene regulations were noticeably downregulated with supplementation of Cu at 8 mg kg^-1^ diet with or without stressors compared to control and other groups. Similarly, gene regulation of *Ig* was remarkably downregulated with As + pH + T and As + T compared to control, and Cu supplemented groups. Further, *Ig* was noticeably upregulated with Cu at 8 mg kg^-1^ diet with or without stressors compared to control and other groups (Fig. 4C).

### Tertiary stress response (Final body weight gain%, FCR, SGR, PER, DGI%, RFI) elevated by non-lethal dose of arsenic, low pH (6.5) and high temperature (34 °C) but dietary copper mitigate it

**Figure 5:**
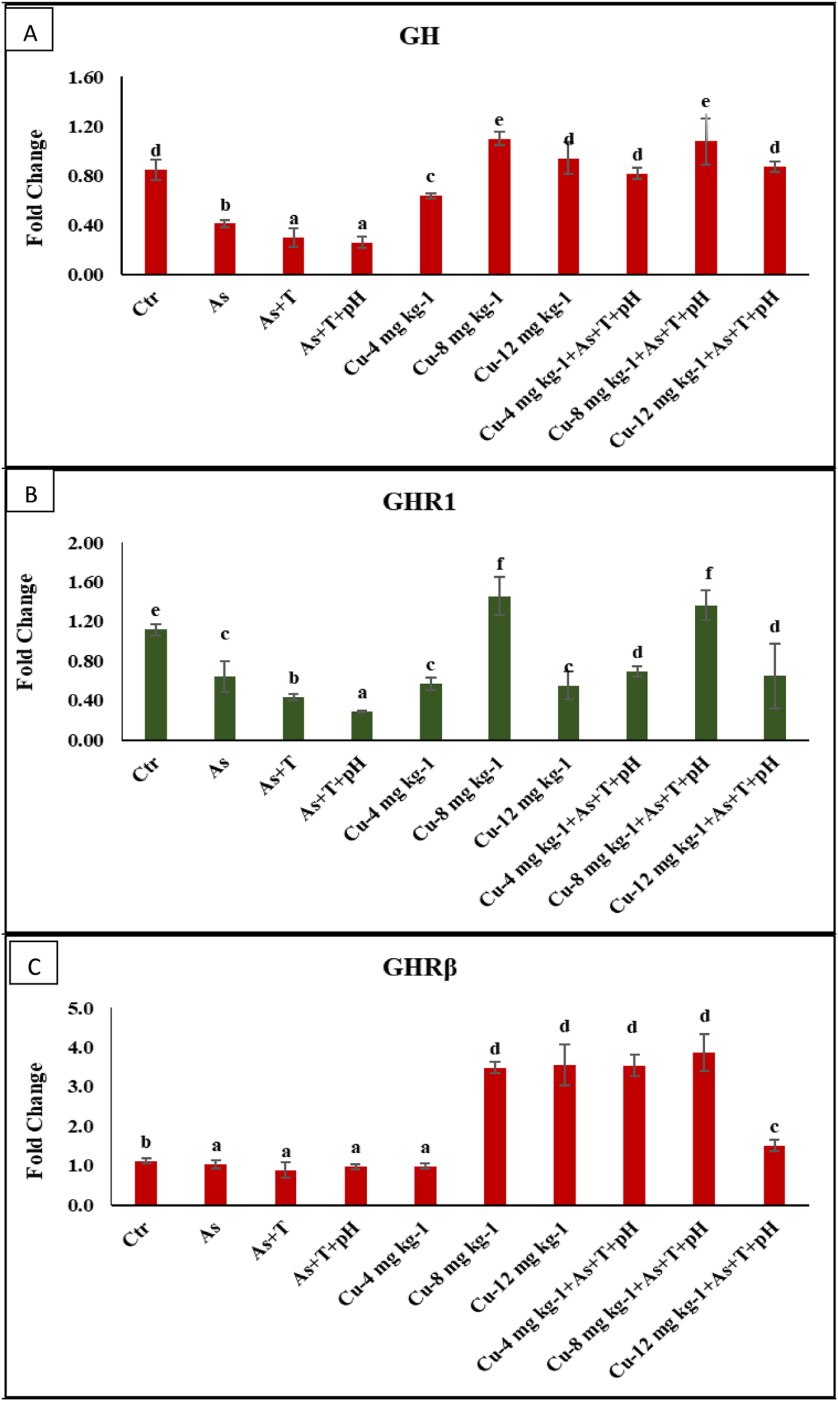
(**A**–**C**) Effect of dietary copper (Cu) on gene expression of GH, GHR1 and GHRβ in *P. hypophthalmus* reared under control or arsenic, low pH (6.5) and high temperature stress (34 °C) for 105 days. Within endpoints and groups, bars with different superscripts differ significantly (**a**–**d**). Data expressed as Mean ± SE (n = 3).

**Figure 6:**
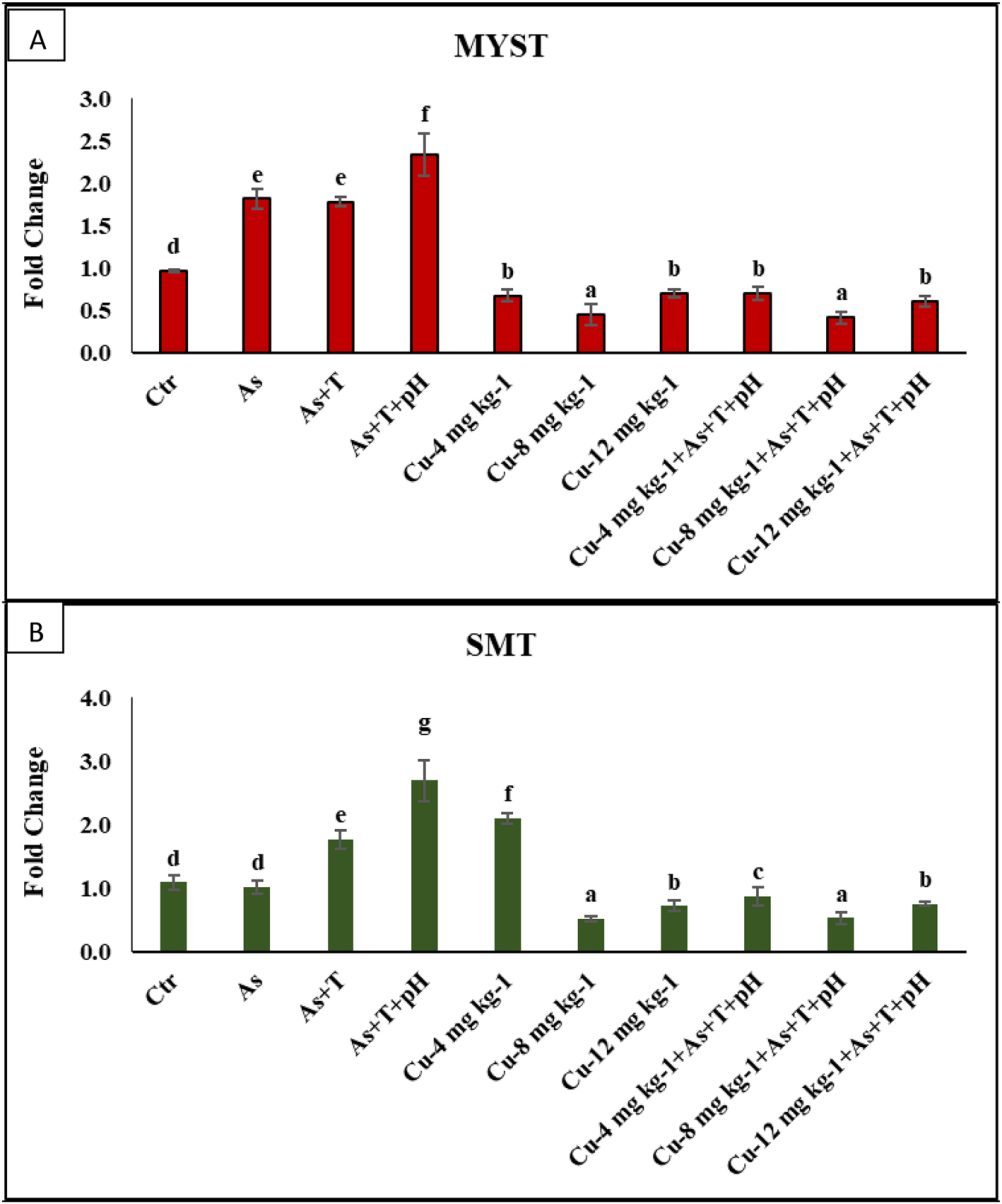
(**A**, **B**): Effect of dietary copper (Cu) on gene expression of MYST and SMT in *P. hypophthalmus* reared under control or arsenic, low pH (6.5) and high temperature stress (34 °C) for 105 days. Within endpoints and groups, bars with different superscripts differ significantly (**a**–**d**). Data expressed as Mean ± SE (n = 3).

Tertiary stress response such as final body weight gain (FBWG%) (*p* = 0.0019), SGR (*p* = 0.0026), PER (*p* = 0.017), DGI% (*p* = 0.025), and RFI (*p* = 0.013) were noticeably reduced with concurrent exposure to low dose arsenic, low pH and high temperature group followed by As + T and As in comparison to control and Cu supplemented groups. Indeed, FBWG%, SGR, PER and DGI%, and RFI were remarkably higher with supplementation of Cu at 8 mg kg^-1^ diet with or without stressors followed by Cu at 4 mg kg^-1^ diet compared to control and other groups. Further, stressors groups (As + pH + T, As + T, and As) significantly enhanced (*p* = 0.0022) the FCR compared to control and Cu supplemented groups. However, the Cu at 8 mg kg^-1^ diet with or without stressors noticeably reduces the FCR, followed by the Cu at 4 mg kg^-1^ diet group compared to control and other groups (Table 8). The gene regulation related to growth performance was determined to evaluate dietary Cu's impact on alleviating a low dose of arsenic, low pH, and high temperature in *P. hypophthalmus*. The genes related to growth performance viz*. GH* (*p* = 0.0045) in liver tissue was noticeably downregulated with As + pH + T and As + T followed by As group, whereas *GHRβ* (*p* = 0.0023) was significantly downregulated with As + pH + T, As + T and As group compared to control and Cu supplemented groups. Moreover, the *GHR1* (*p* = 0.0039) was remarkably downregulated with As + pH + T followed by As + T and As group compared to control and other groups. Indeed, *GH, GHR1*, and *GHRβ* gene regulation was noticeably upregulated with Cu at 8 mg kg^-1^ diet with or without stressors compared to control and other groups. The other Cu supplemented diets (Cu 4 and 12 mg kg^-1^ diet) were not improved the genes regulation involved in growth performance (Fig. 5A–C). In addition, the *MYST* (*p* = 0.0011) and *SMT* (*p* = 0.0042) genes were noticeably downregulated with Cu supplementation at 8 mg kg^-1^ diet with or without stressors groups compared to the control and exposure group. Exposure to As + pH + T followed by As + T upregulated *MYST* and *SMT* gene regulations compared to control and other groups (Fig. 6A, B).

**Table 8:** Effect of dietary copper (Cu) to improve the final body weight gain%, FCR, SGR, PER, DGI, TGC and RFI in *P. hypophthalmus* reared under control or arsenic, low pH (6.5) and high temperature (34 °C) stress for 105 days.

Treatment	Non-Stressors	Stressors			Non-Stressors			Stressors (As + T + pH)			*p*-Value
		As	As + T	As + T + pH	Cu-4.0 mg kg^-1^	Cu-8.0 mg kg^-1^	Cu-12.0 mg kg^-1^	Cu-4.0 mg kg^-1^	Cu-8.0 mg kg^-1^	Cu-12.0 mg kg^-1^	
Diet	Control	Control	Control	Control							
Final body weight gain (%)	133.88e ± 5.69	89.31^c^ ± 4.67	81.00b ± 0.93	66.41a ± 2.15	145.84f. ± 8.32	215.40 g ± 2.36	114.95d ± 4.12	153.09f. ± 12.63	214.52 g ± 4.34	116.50d ± 5.58	0.0019
FCR	2.53^c^ ± 0.09	3.38^e^ ± 0.14	3.60f. ± 0.04	4.28 g ± 0.11	2.39b ± 0.10	1.91a ± 0.01	2.88d ± 0.09	2.30b ± 0.13	1.93a ± 0.02	2.89d ± 0.10	0.0022
SGR	0.72c ± 0.03	0.56b ± 0.04	0.52b ± 0.01	0.45a ± 0.01	0.77c ± 0.03	1.00e ± 0.01	0.70c ± 0.01	0.80d ± 0.04	1.05e ± 0.01	0.74c ± 0.01	0.0026
PER	1.14d ± 0.06	0.96c ± 0.04	0.86b ± 0.01	0.73a ± 0.03	1.30e ± 0.07	1.66f. ± 0.03	1.06d ± 0.04	1.35e ± 0.08	1.58f. ± 0.02	1.06d ± 0.04	0.017
DGI (%)	1.16c ± 0.04	0.86b ± 0.03	0.80b ± 0.01	0.68a ± 0.02	1.30d ± 0.05	1.72e ± 0.01	1.07c ± 0.03	1.34d ± 0.07	1.70e ± 0.02	1.06c ± 0.03	0.025
TGC	0.04 ± 0.00	0.04 ± 0.00	0.03 ± 0.00	0.03 ± 0.00	0.04 ± 0.00	0.04 ± 0.00	0.04 ± 0.00	0.03 ± 0.00	0.03 ± 0.00	0.03 ± 0.00	0.081
RFI	337.45^e^ ± 3.01	300.15^c^ ± 3.44	291.80^b^ ± 1.08	283.67^a^ ± 1.92	347.61^e^ ± 5.72	410.90^ g^ ± 5.60	329.77^d^ ± 1.59	348.72^e^ ± 9.17	415.03^ g^ ± 3.39	335.35^e^ ± 4.84	0.013

Values in the same row with different superscript (a, b, c, d, e) differ significantly. Data expressed as Mean ± SE (n = 3).

FCR feed conversion ratio, SGR specific growth rate, PER protein efficiency ratio, DGI Daily growth index, TGC Thermal growth coefficient, *RFI* relative feed intake.

### Arsenic bioaccumulation

Arsenic bioaccumulation in different fish tissues such as liver, gill, muscle, brain, and kidney were determined at the end of the experiment. In addition to tissues, As concentration in the water sample and Cu content in fish muscle was also determined. Results revealed that the highest bioaccumulation was determined in the liver tissues, followed by kidney, gill, brain, and muscle in the group exposed to As + pH + T. The group fed with Cu at 4, 8, and 12 mg kg^-1^ diet without stressors were not detectable arsenic in brain and muscle tissues. However, the group exposed to stressors (As + pH + T) and fed with a Cu diet at 8 mg kg^-1^ was determined the lowest bioaccumulation in all the tissues. The same pattern was obtained in the case of arsenic in water samples. In the case of Cu content in the muscle tissue, the highest Cu was obtained in the group fed with 12 mg kg^-1^ diet with or without stressors (Table 9).

**Table 9:** Dietary copper reduced the arsenic concentration in water and bioaccumulation in different tissues of *P. hypophthalmus* reared under control or arsenic, low pH (6.5) and high temperature stress (34 °C) for 105 days.

Treatments	Non-Stressors	Stressors			Non-Stressors			Stressors (As + T + pH)		
		As	As + T	As + T + pH	Cu-4.0 mg kg^-1^	Cu-8.0 mg kg^-1^	Cu-12.0 mg kg^-1^	Cu-4.0 mg kg^-1^	Cu-8.0 mg kg^-1^	Cu-12.0 mg kg^-1^
Diet	Control	Control	Control	Control						
Water	0.79 ± 0.10	1321.73 ± 66.45	1568.74 ± 45.52	1738.80 ± 29.03	0.04 ± 0.01	0.03 ± 0.01	0.11 ± 0.01	883.92 ± 39.81	361.81 ± 48.28	1187.07 ± 87.66
Liver	0.57 ± 0.07	8.98 ± 0.14	11.56 ± 0.61	14.37 ± 0.38	0.20 ± 0.02	0.06 ± 0.01	0.24 ± 0.11	5.75 ± 0.50	1.72 ± 0.56	9.31 ± 0.74
Gill	0.16 ± 0.02	6.65 ± 0.25	8.49 ± 0.10	9.36 ± 0.14	0.04 ± 0.01	0.01 ± 0.00	0.06 ± 0.01	2.31 ± 0.16	0.94 ± 0.04	3.12 ± 0.09
Kidney	0.95 ± 0.03	10.79 ± 0.43	12.22 ± 0.48	13.25 ± 0.35	0.09 ± 0.01	0.01 ± 0.00	0.07 ± 0.01	6.72 ± 0.23	1.96 ± 0.17	8.78 ± 0.25
Brain	0.00 ± 0.00	2.55 ± 0.05	3.08 ± 0.06	3.37 ± 0.06	ND	ND	ND	1.40 ± 0.09	0.86 ± 0.07	2.04 ± 0.08
Muscle	0.00 ± 0.00	0.41 ± 0.03	0.57 ± 0.03	0.92 ± 0.04	ND	ND	ND	0.45 ± 0.32	0.04 ± 0.01	0.94 ± 0.02
Muscle-Cu	0.66 ± 0.05	0.43 ± 0.12	0.65 ± 0.05	0.75 ± 0.07	1.38 ± 0.13	2.35 ± 0.45	8.24 ± 0.40	2.46 ± 0.26	1.05 ± 0.02	3.99 ± 0.30

ND Below detection limit, Data expressed as Mean ± SE (n = 3). Concentration of arsenic in water (µg L^-1^), liver, gill, kidney, brain and muscle (mg kg^-1^).

### Bacterial infection to fish (cumulative mortality and relative survival%) reared under non-lethal dose of arsenic, low pH (6.5) and high temperature (34 °C)

**Figure 7:**
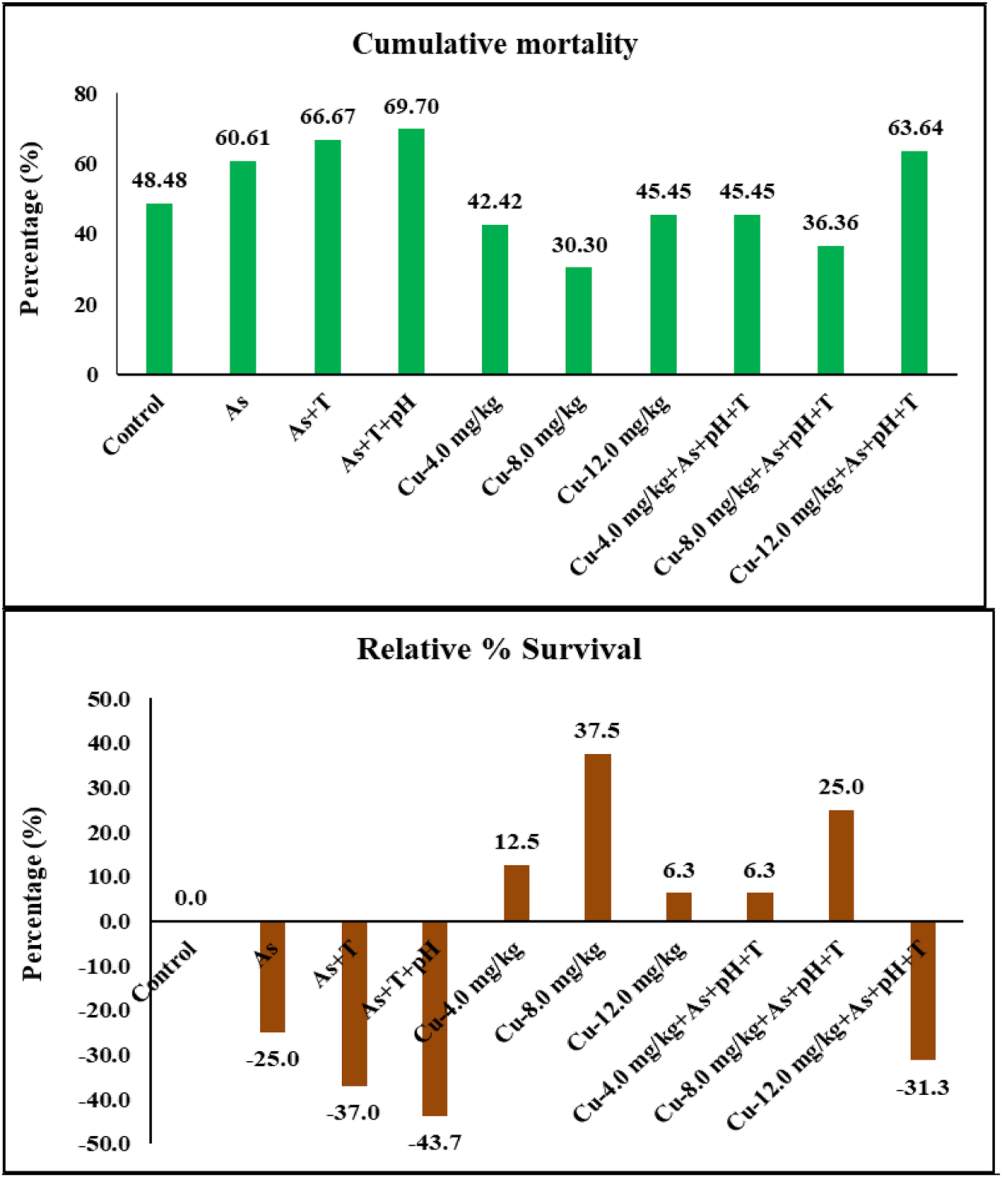
Effect of dietary copper (Cu) on cumulative mortality and relative percentage survival after *A. hydrophila* infection in *P. hypophthalmus* reared under control or arsenic, low pH (6.5) and high temperature stress (34 °C) for 105 days.

Total of 33 fish in each treatment were (11 fish in each replicate) injected with *Aeromonas hydrophila* after 105 days of experimental periods and observed the cumulative mortality and relative survival (%) for 7 days. The cumulative mortality was observed as 48, 60, 66, 69, 42, 30, 45, 45, 36, and 63% in control, As, As + T, As + pH + T, Cu at 4, 8 and 12 mg kg^-1^ diet with or without stressors respectively. Similarly, relative survival (%) was observed as 0, 25, 37, 43, − 12, − 37, − 6, − 6, − 25 and 31% in respective group as above (Fig. 7).

## Discussion

The present investigation focused on addressing the impact of heavy metals, particularly arsenic, as well as low pH and high-temperature stress in aquaculture. These abiotic factors significantly affect the physiological, biochemical, and molecular processes in fish. This study also examined various gene regulation mechanisms responsible for inducing stress in fish. Cortisol is the primary stress hormone which secreted from hypothalamus–pituitary–interrenal (HPI) axis, and released the glucocorticoids from interrenal cells in the kidney tissues of the fish^64^. It maintained the physiological process, behavioral adjustments, and adaptive metabolism in fish^65^. In the present investigation, the stressors (As + pH + T) interfere with the biosynthesis and synthesis of cortisol using the hypothalamus–pituitary–interrenal (HPI) axis resulting in altered cholesterol synthesis and adrenocorticotrophic hormone. Cortisol's inactive form, cortisone, is converted to its active form, cortisol, through the catalysis 11 β-hydroxysteroid dehydrogenases. In case of exposed to stress, the expression of CRH-BP increases in a time-dependent manner. This increase is believed to function as a negative feedback mechanism to reduce the interaction of CRH with CRH-R1^66^. Moreover, the supplementation of Cu at 8 mg kg^-1^ diet potentially reduces the cortisol level due to its role in several enzyme as a co-factor such as lysyl oxidase, cytochrome c oxidase, ceruloplasmin, and dopamine monooxygenase.

*CYP 450* is the hemeprotein involved in the metabolism of xenobiotics and other drugs^67^. It is also beneficial for mono-oxygenation reactions of many exogenous and endogenous compounds in fish, animals, humans, and plants. *CYP 450* is essential in endogenous detoxifying substrates, viz. fatty acids, vitamins, prostanoids, and steroids. Moreover, in exogenous substrates such as chemicals, drugs, metals, and pesticides^68^. The results revealed that *CYP 450* was upregulated by concurrent exposure to As + pH + T and other different stressors combinations. The results reflected that detoxification of arsenic was enhanced in the different fish tissues which showed that *CYP 45*0 could not support the detoxication in the fish tissues. Surprisingly, the supplementation of Cu at 8 mg kg^-1^ diet significantly downregulated the *CYP 450* gene regulation. At the same time, the detoxification of arsenic was lowest in the group fed with Cu at 8 mg kg^-1^ diet. These results revealed that Cu supports the arsenic detoxification by activating *CYP 450* gene regulation. It has also come across that copper activated the NF-κB signaling pathway and regulated the *CYP 450* gene expression with nuclear receptors using mutual suppression^69^. *Cas 3a* is a crucial member of apoptosis, belong to cysteine proteases, and play a vital role in multiple physiological processes such as immunity and development of the organism and process like transduction, induction, amplification of intracellular apoptotic signals, and transduction process^70,71^. It also has a critical role in removing damaged tissues or unwanted cells without affecting the other tissues^71,72^. In the present investigation, arsenic, low pH, and high-temperature stress upregulated the *Cas 3* gene expression due to the removal or targeting of the damaged tissues. However, due to excessive damage to tissues, the *CAS 3a* gene might be accumulated and upregulated the gene expression. Surprisingly, the supplementation of Cu at 8 mg kg^-1^ diet was noticeably downregulated the *Cas 3a* gene expression. It might be because of Cu enhances the removal of damaged tissues quickly. However, the gene expression was downregulated in the Cu-fed group (8 mg kg^-1^ diet). Any research did not report such a mechanistic role of Cu in the downregulation of the *Cas 3a*. This is the first report that Cu mechanistic role in *Cas 3a* gene regulation in fish reread under multiple stressors (As + pH + T). *MT* genes are ubiquitous, high cysteine, low molecular weight stress-induced protein possessing protection against different metal toxicity, diverse physiological function, role in apoptosis, and regulation of cellular proliferation in fish and other living organisms^73,74^. In the present investigation, the stressors groups (As + pH + T) were noticeably elevated *MT* gene expression, which might be due to bond formation between stressors groups and the *MT* gene, especially arsenic^75^. Moreover, the dietary Cu groups (4 and 8 mg kg^-1^ diet) were remarkably downregulated MT gene expression. It could be due to Cu help to the *MT* gene to release the thiol group and multiple cysteines, so it reacts with oxidant^76^.

HSPs are highly conserved molecular chaperon proteins that function as brain machinery to control oxidative challenges and are upregulated during stress^77^. *HSPs 70* gene expression is generally upregulated during temperature stress^2,12,13^ and metal stress^62,78^ and considered as a potent biomarker for stress response in fish. Similarly, in this study, the exposure to arsenic, low pH, and high temperature upregulated the *HSP 70* in liver tissues. Interestingly, the supplementation of Cu diet (8 and 4 mg kg^-1^ diet) noticeably downregulates the *HSP 70* gene expression and mitigates As + pH + T stress in fish. Indeed, the Cu-containing diet as nutriments downregulating the *HSP 70* expression could be due to its role in ApoA-1 gene regulation. Not much information is available on the role of dietary Cu in HSP expression of fish. Similarly, the gene expression of iNOS was upregulated by stressors (As, As + T and As + pH + T) in *P. hypophthalmus* whereas the Cu diet at 8 mg kg^-1^ diet downregulated the *iNOS* gene expression. This could be due to the feed intake of fish being noticeably reduced in stressors groups and build-up ammonia. This is probably the reason for the upregulation of the *iNOS* gene. Moreover, the Cu supplementation downregulated the *iNOS* gene expression that which could be due to Cu enhancing the absorption or utilization of ammonia. Still, we could not find such report. Moreover, the DNA damage (comet area, comet length, comet DNA, head area, head DNA, head DNA%, tail area, tail DNA, and tail DNA%) was noticeably higher in groups treated under stressors (As, As + T and As + pH + T). Surprisingly, the Cu diet (4 and 8 mg kg^-1^ diet) protects against DNA damage due to its role in ceruloplasmin, tyrosinase, and dopamine hydroxylase. The study by Webster et al.^79^ proposed that feeding with a low Cu diet protects against DNA damage in Rat. However, it is also revealed that low Cu is necessary to maintain the structural integrity of DNA during oxidative stress.

The stressors such as As, As + T, and As + pH + T upregulated the fish's *CAT, SOD*, and *GPx* gene regulation and biochemical activities. Arsenic is toxic and denatures the cellular anti-oxidant system resulting in a higher inflammation rate and accumulated free radicals in the cell system^80,81^. Low pH also induces stress and generates ROS. It interferes with biological molecules as DNA or other signaling molecules^82^. Low pH enhances the transcription of ferritin, a member of the protein family (orchestrates), which protect the cellular defense against stress^83^. However, the low pH also affects oxygen affinity and consumption, which is reflected in the enhancement of oxidative stress ions in the present investigation. Indeed, the Cu diet protect the tissues against stressors could be due to its role in anti-oxidant defence using cytochrome c oxidase (energy production), tyrosinase, lysyl oxidase (extracellular matrix protein crosslinking), dopamine-b-hydroxylase, peptidylglycine alpha-amidating monooxygenase, monoamine oxidase (pigment and neurotransmitter production and metabolism), copper-zinc superoxide dismutase (Cu–Zn-SOD; SOD1) and ceruloplasmin (ferroxidase activity)^84^. LPO in the liver, gill, and kidney was noticeably reduced with supplementation of the Cu diet, which maintained the enzymatic co-factors as Cu–Zn-SOD and maintained mitochondrial Fe–sulfur proteins to control LPO in the tissues^85^.

ALT and AST are important biomarkers enzymes for tissue damage due to pollutants and other stress^86^. In this study, the arsenic, low pH, and high-temperature stress elevated the ALT and AST activities. This could be due to tissue damage, especially liver, due to stress^86^. Indeed, the ALT and AST were noticeably reduced with supplementation of the Cu diet could be due to the detoxification of pollutant rate enhanced by Cu^87^. In the present study, the LDH and MDH activities were altered with exposure to arsenic, low pH, and high temperature, whereas dietary Cu corrected the activities. LDH and MDH are important biomarkers widely used in toxicity studies^86^. However, during stress, the fish might be needed more energy demand to fulfil all the requirements. For that, dietary Cu could support energy demand and release stress in animals and fish. Vitamin C in muscle and brain and AChE activities in brain was remarkably inhibited due to exposure to As + pH + T. It could be due to the brain's high vulnerability to As, which possesses a high oxygen consumption rate and polyunsaturated fatty acids. Resulting in the generation of high rate of oxygen free radical without a commensurate level of As^88^ and inhibiting the cholinergic system in the brain and forming cyclic dithioarsenite diester^2,89^. Surprisingly, the Cu diet improved the AChE and Vit C due to the role of Cu in homeostasis and neuropathological^90,91^. Cu also enhanced the vitamin C level in fish's brain and muscle tissue. It is an essential component for collagen synthesis and crucial in the metabolism of steroids and detoxification of xenobiotics^92^.

In the present investigation, supplementation of Cu enhances MPO could be due to stimulation of B-lymphocytes which enhanced the cytokines released from macrophages or other phagocytes. Moreover, albumin is essential for transporting hormones, metals, drugs, vitamins, fat metabolites, and bilirubin and regulates the free available hormones^32^. The high energy demand during stress was fulfilled by albumin via protein synthesis. On the other side, the nitro blue tetrazolium (NBT) determined the functioning of phagocytes and a higher NBT level indicates higher non-specific immunity^93^. However, the fish immune systems may be damaged by harmful chemicals or stressed by free radicals and pro-oxidants but at an optimum level, Cu diet acts as co-factor of specific protein and enzymes such as ceruloplasmin and superoxide dismutase^94^.

The exposure to As, As + T and As + pH + T altered the *Ig,* and *TNFα* gene expression and weaken the immunity of the *P. hypophthalamus*. The results of downregulation of *Ig* gene and upregulation of *TNFα* gene expression showed weak immunity. Indeed, the Cu-containing diet corrected *Ig,* and *TNFα*, gene expression, indicating strong immunity. Generally, *TNFα* is considered pro-inflammatory cytokines, which are important markers to evaluate inflammatory response during immune system stimulus^95^. *TNFα* is produced through the T-cells of macrophages. However, supplementation of the dietary Cu diet prevents the liver tissues from the inflammatory response. This investigation is the first report that dietary Cu protects the tissues from the inflammatory response and enhances the fish's immunity against multiple stresses.

The water qualities affect the growth performance of the fish. The exposure to arsenic, low pH, and high temperature noticeably reduced the growth performance (final body weight gain%, FCR, SGR, PER, DGI%, TGC, and RFI) could be due to decreases in feed intake and metabolic rate, which was reported by our previous study^12,13^. Surprisingly, the supplementation of the Cu diet remarkably enhances the growth performance, which could be due to its role in the enhancement of feed efficiency, feed utilization, growth rate, and immunity of the fish. It also improves the protein efficiency, specific growth rate, daily growth index, and relative feed intake in the fish. *GH, GHR1, GHRβ, MYST*, and *SMT* were noticeably improved by Cu containing diet. *GH* may be binding to GHR, which plays an essential role in regulating growth and development. The secretion of *GH* is under the control of hypothalamic regulation by modulators such as somatostatin, GH-releasing hormone, dopamine, and ghrelin^96,97^. Generally, growth is genetically regulated and affected by cellular, endocrinological and environmental factors in which the endocrinal tissues are affected by the integration of external stimuli and internal signal based on physiological status^97^. However, the growth can be performed better with suitable nutrition, optimum temperature, good husbandry condition, and changes in the endocrine systems of the animal^98^ [93]. *GH* plays a vital role in regulating critical physiological phenomena viz. osmotic balance, growth, and strengthening the immunity. The exposure to different stressors As, As + T, and As + pH + T significantly reduced the growth rate as *GH*, *GHR1* and GHRβ expression were drastically decreased^98,99^ This might be due to the interaction of growth-related genes and glucocorticoids^100^. However, *SMT* and *MYST* gene expression has been noticeably upregulated by stressors in the present investigation, whereas the supplementation of Cu at 8 mg kg^-1^ diet downregulated the expression of *SMT* and *MYST* gene. Generally, *MYST* depresses the myoblast, which results in terminal differentiation and division of fiber enlargement^101^.

The dietary Cu reduces arsenic bioaccumulation in different fish tissues because of the role of Cu in enhancing arsenic detoxification. Indeed, the highest arsenic bioaccumulation was observed in the liver tissue, followed by kidney tissues. Research on dietary copper and arsenic removal is very much scanty. The mechanism behind the low bioaccumulation of arsenic could be due to the enhancement of rate of arsenic assimilation and detoxification by target organs using Cu diet.

The results revealed that exposure to As, As + T, and As + pH + T reduces the immunity of the fish. However, the bacterial infection enhances the cumulative mortality and relative survival (%) in *P. hypophthalmus*. Our earlier reports demonstrated that multiple stresses enhance bacterial infection^12,13^. Interestingly, Cu has not only an essential element but also a potent immunomodulator in aquatic animals^102^. Therefore, the Cu diet reduces the mortality against bacterial infection. Moreover, deficiency of Cu reduces cell-mediated, non-specific, and humoural immunity^103^.

## Conclusions

The present investigation addressed the prominent issues of aquaculture and fisheries-related to climate change and pollution in aquatic systems. Over the last two-decade, many fish species have been extinct due to sudden changes in climate and the level of pollution present in the aquatic systems. Moreover, this study showed that supplementation of Cu-containing diet, mainly 8 mg kg^-1^ followed by 4 mg kg^-1^ diet, mitigates the primary, secondary and tertiary stress response. The present study also revealed that Cu-containing diets improve the gene expression involved in climate change and pollution. The mechanistic role of Cu using several genes witnessed the improvement of the well-being of the fish in such drastic conditions (As, As + T and As + pH + T) and produced contaminated free fish production. Indeed, the results of the present study revealed that Cu at 8 mg kg^-1^ diet improved the well-being and extinction of the fish species in the recent climate and pollution era.

### Supplementary Information

Supplementary Information.

## Data Availability

The datasets generated during and/or analyzed during the current study are available from the corresponding author on reasonable request.

## Abbreviations

Cu: Copper
As: Arsenic
T: High temperature
*NFkB*: Nuclear factor kappa B
*iNOS*: Inducible nitric oxide synthase
*HSP70*: Heat shock protein
*MT*: Metallothionine
*CAT*: Catalase
*SOD*: Superoxide dismutase
*GPx*: Glutathione peroxidase
*CA**S **3a*: Caspase 3a
*TNFα*: Tumor necrosis factor
CRH: Corticotropin-releasing hormone
CRH-BP: Cortisol releasing hormone binding protein
CRH-R1: Cortisol releasing hormone receptor 1
*TLR*: Toll-like receptors
*GH*: Growth hormone
*GHR1; GHRβ*: Growth hormone regulator 1 and β
*MYST*: Myostatin
*SMT*: Somatostatin
IPCC: Intergovernmental panel on climate change
TAN: Total ammonia nitrogen
RAC: Research advisory committee
PME: Prioritization, monitoring and evaluation
LC_50_: Lethal concentration
CMC: Carboxymethyl cellulose
CP: Crude protein
EE: Ether extract
FEC: Feed conversion efficiency
SGR: Specific growth rate
PER: Protein efficiency ratio
DGI: Daily growth index
TGC: Thermal growth coefficient
RFI: Relative feed intake
ICPMS: Inductively coupled plasma mass spectrometry
