# Supplementary material for: Multifunctional role of dietary copper to regulate stress-responsive gene for mitigation of multiple stresses in Pangasianodon hypophthalmus
Source: Sci Rep. 2024 Jan 26;14:2252. doi: 10.1038/s41598-024-51170-z (PMC10817903; doi:10.1038/s41598-024-51170-z)
Supplement: Supplementary file 1 — Supplementary Information. [file 41598_2024_51170_MOESM1_ESM.doc]

Supp Table 1: Physico-chemical parameters of water during the experimental period of 105 days

| Treatments | Temperature (°C) | pH | DO (mgL^-1^ ) | Hardness (mgL^-1^ ) | Ammonia-N (mgL^-1^ ) |
| --- | --- | --- | --- | --- | --- |
| Ctr | 26.1-28.20 | 7.4-7.7 | 6.2-7.1 | 224.2-229.4 | 0.11-0.12 |
| As | 25.5-27.78 | 7.3-7.6 | 6.3-7.2 | 219.1-223.5 | 0.12-0.14 |
| As+T | 34.12-35.17 | 7.1-7.8 | 6.1-6.8 | 221.2-229.6 | 0.10-0.11 |
| As+T+pH | 34.35-34.92 | 6.3-6.5 | 6.2-6.7 | 222.2-228.4 | 0.13-0.15 |
| Cu-4 mg kg^-1^ | 26.4-28.6 | 7.6-7.7 | 6.3-6.9 | 224.1-228.3 | 0.12-0.14 |
| Cu-8 mg kg^-1^ | 24.6-29.1 | 7.2-7.4 | 6.4-7.2 | 224.3-228.2 | 0.10-0.12 |
| Cu-12 mg kg^-1^ | 25.5-28.2 | 7.3-7.6 | 6.5-6.9 | 221.5-227.2 | 0.11-0.13 |
| Cu-4 mg kg^-1^+As+T+pH | 33.8-34.8 | 6.2-6.4 | 6.1-6.4 | 218.1-219.1 | 0.12-0.13 |
| Cu-8 mg kg^-1^+As+T+pH | 34.7-35.1 | 6.1-6.5 | 6.0-6.4 | 217.3-221.4 | 0.14-0.16 |
| Cu-12 mg kg^-1^+As+T+pH | 34.7-35.1 | 6.3-6.5 | 6.2-6.5 | 222.3-227.2 | 0.10-0.13 |

Supp Table 2: The PCR efficiency of all the genes

| S. No | Genes | PCR Efficiency |
| --- | --- | --- |
|  | Cytochrome P 450 (*CYP 450*) | 1.4909 |
|  | Caspase 3a (*Cas 3a*) | 1.3652 |
|  | Metallothionine (*MT*) | 1.5462 |
|  | Heat shock protein (*HSP70*) | 1.3530 |
|  | Inducible nitric oxide synthase (*iNOS*) | 1.3906 |
|  | Superoxide dismutase (*SOD*) | 1.3862 |
|  | Catalase (*CAT*) | 1.3897 |
|  | Glutathione peroxidase (*GPx*) | 1.4381 |
|  | Tumor necrosis factor (*TNFα*) | 1.4165 |
|  | Toll like receptor (*TLR*) | 1.3935 |
|  | Total immunoglobulin (*Ig*) | 1.3747 |
|  | Growth hormone (*GH*) | 1.3572 |
|  | Growth hormone regulator 1 (*GHR1*) | 1.3968 |
|  | Growth hormone regulator β (*GHRβ*) | 1.4228 |
|  | Myostatin (*MYST*) | 1.4363 |
|  | Somatostatin (*SMT*) | 1.4113 |

Supp Figure 1: The details of efficiency and the standard curve of *CYP 450, Cas 3a, MT, HSP 70,* and *iNOS*

| 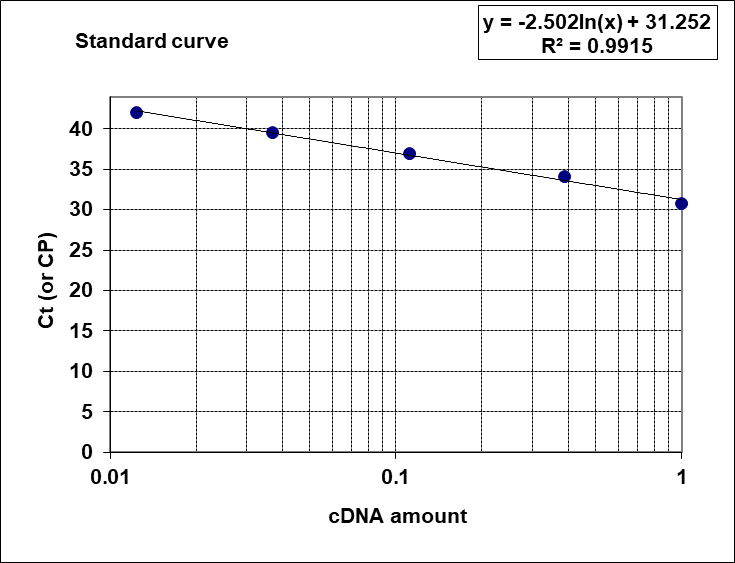 | 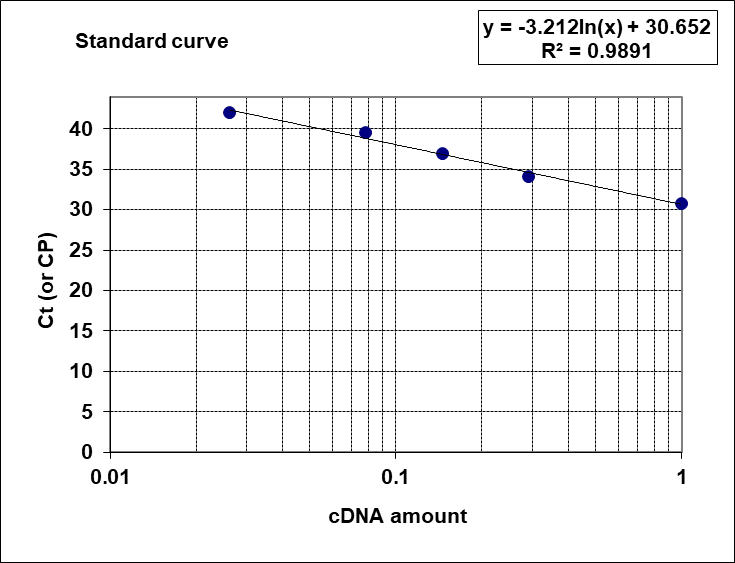 | 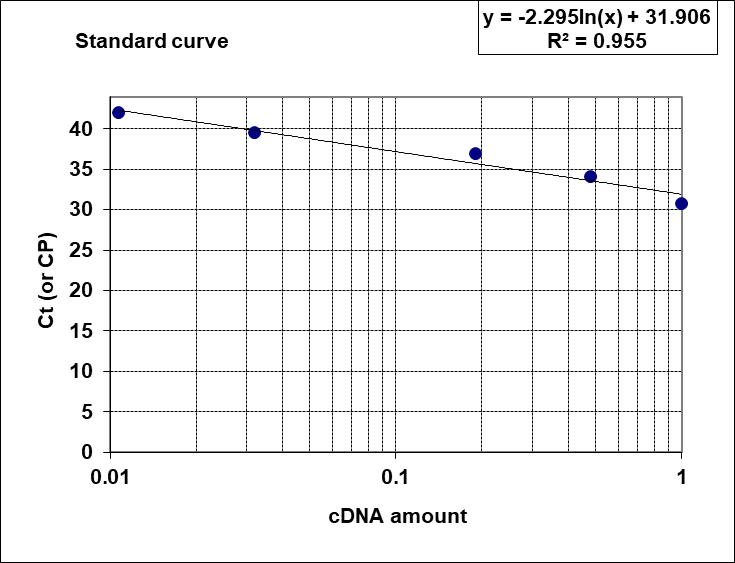 |
| --- | --- | --- |
| *CYP 450* | *Cas 3a* | *MT* |
| 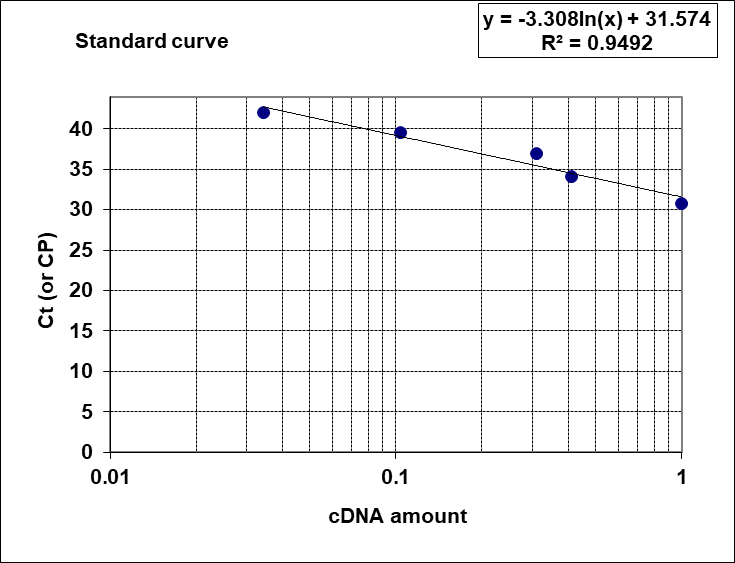 | 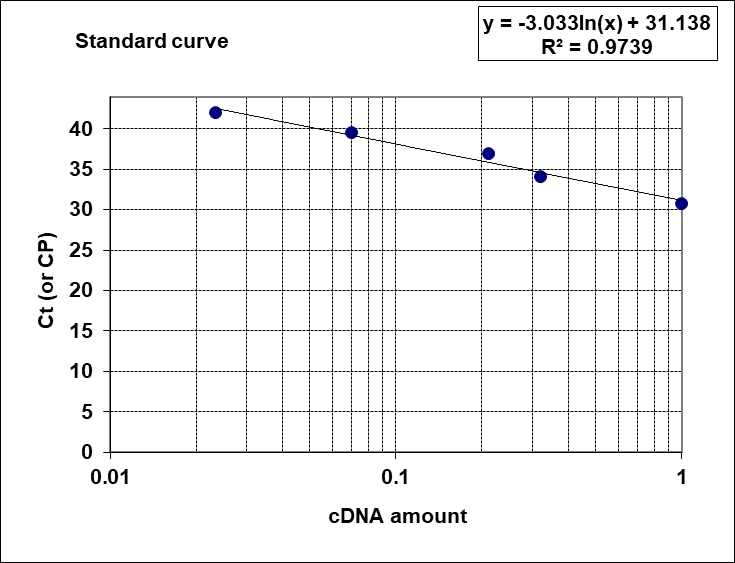 |  |
| *HSP 70* | *iNOS* |  |

Supp Figure 2: The details of efficiency and the standard curve of *SOD, CAT, GPx, TNFα, and TLR*

| 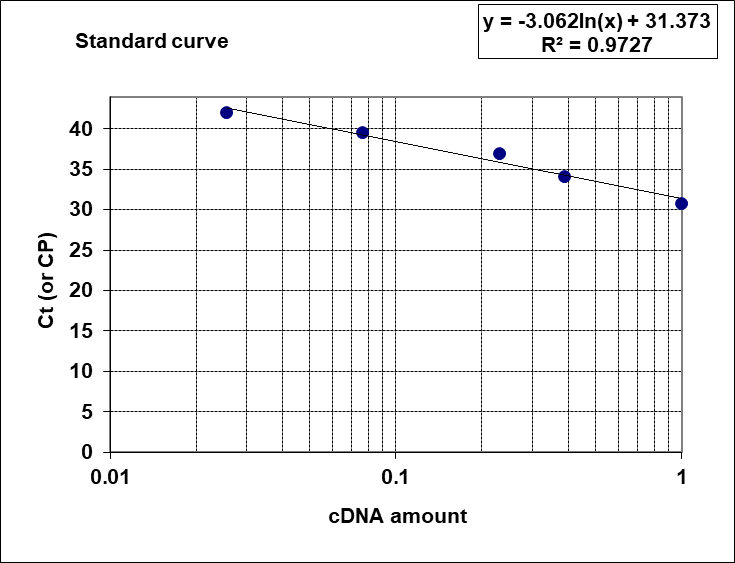 | 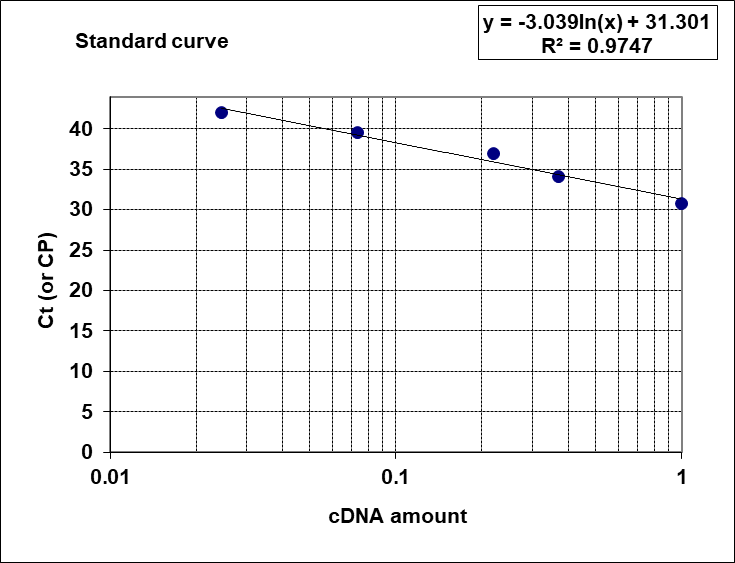 | 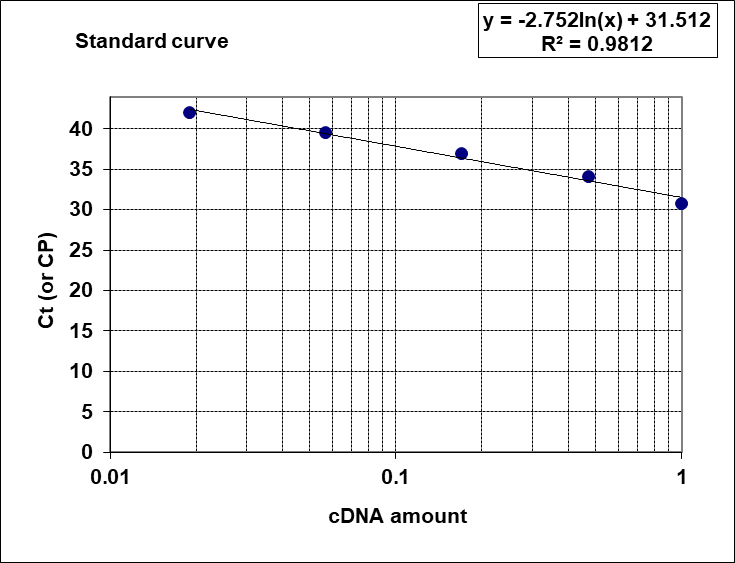 |
| --- | --- | --- |
| *SOD* | *CAT* | *GPx* |
| 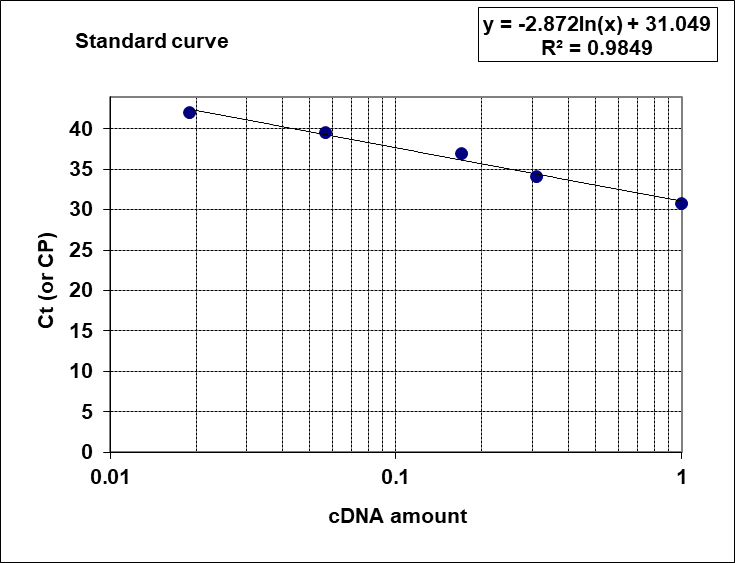 | 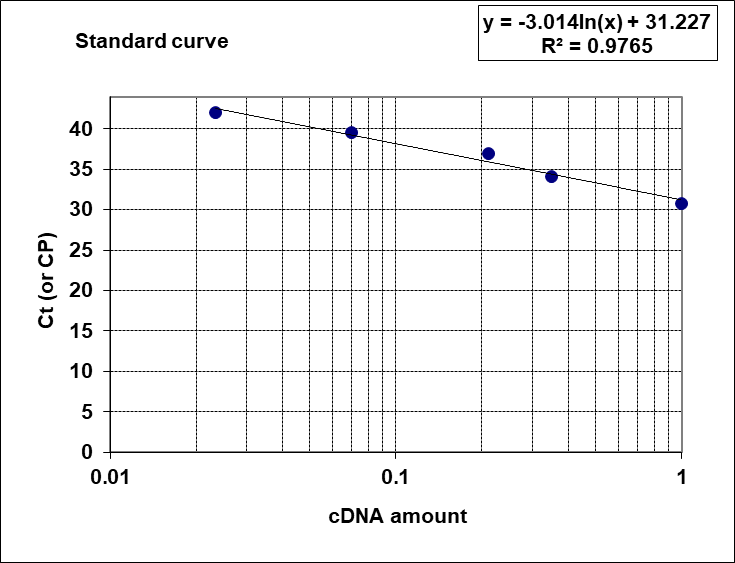 |  |
| *TNFα* | *TLR* |  |

Supp Figure 3: The details of efficiency and the standard curve of *Ig, GH, GHR1, GHRβ, MYST* and *SMT*

| 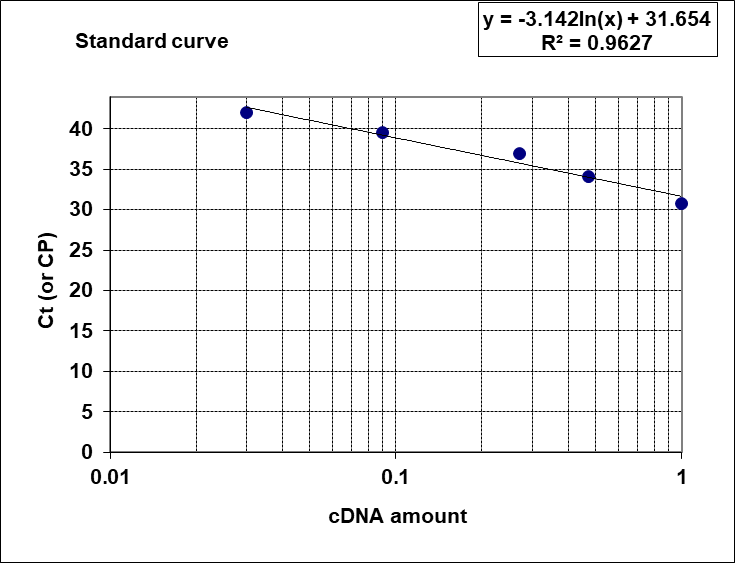 | 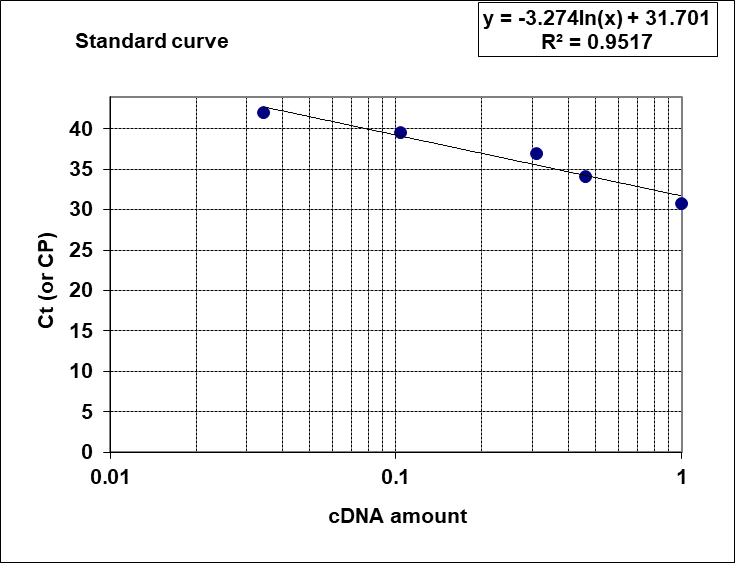 | 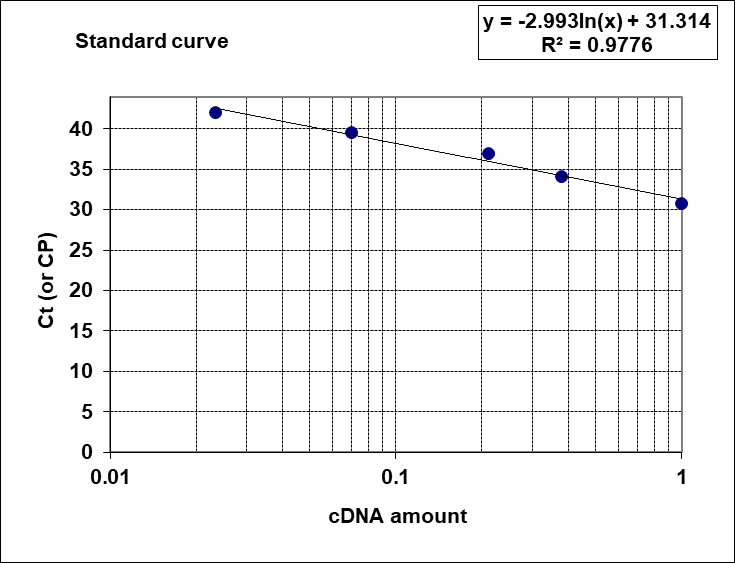 |
| --- | --- | --- |
| *Ig* | *GH* | *GHR1* |
| 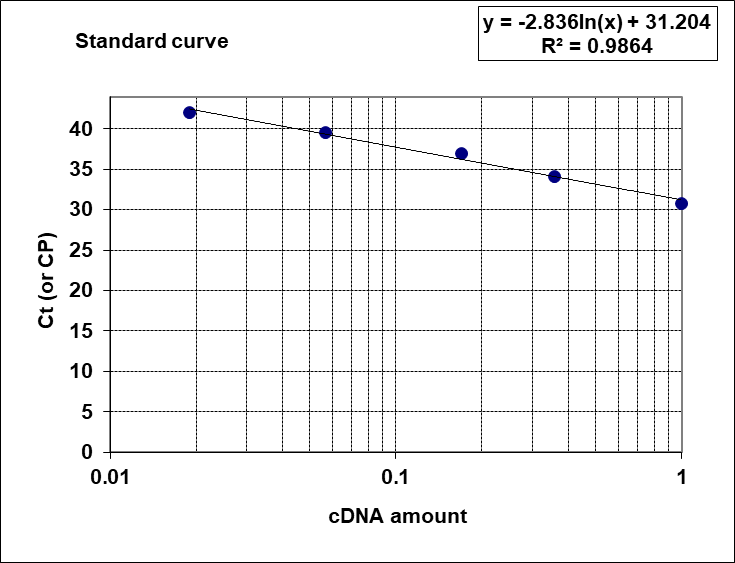 | 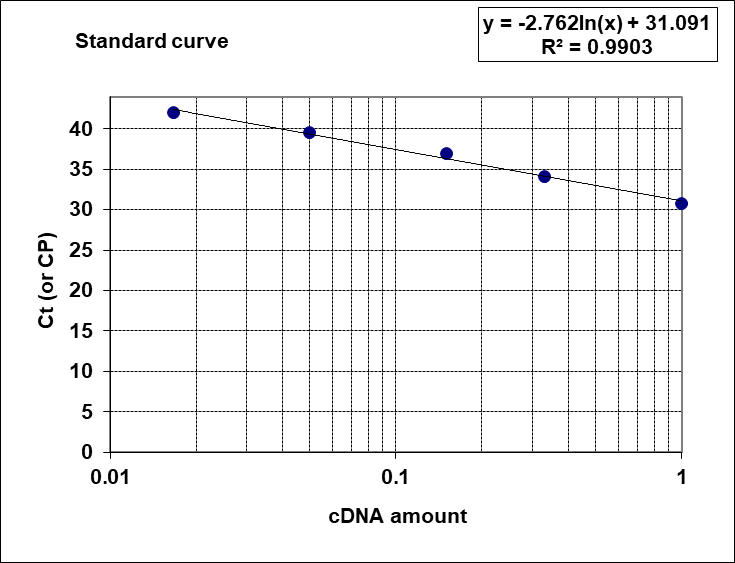 | 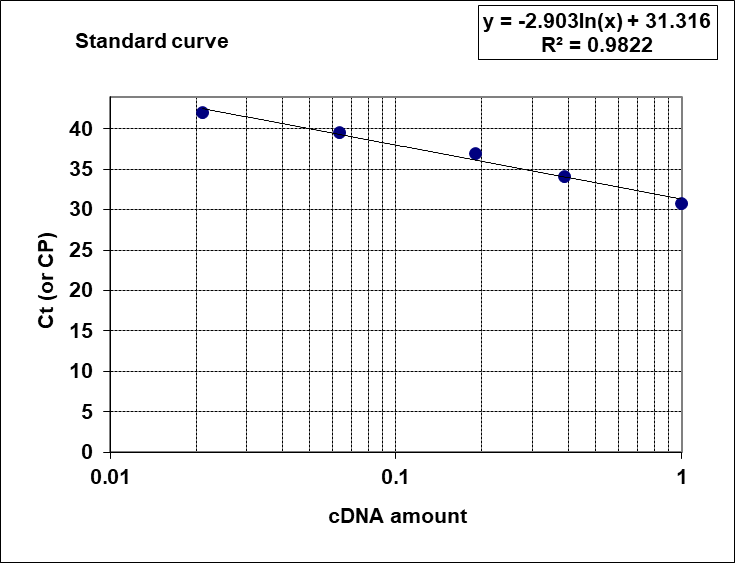 |
| *GHRβ* | *MYST* | *SMT* |
